# FoxO transcription factors coordinate the urea cycle and gluconeogenesis by controlling *Ass1*

**DOI:** 10.1016/j.isci.2026.117399

**Published:** 2026-08-31

**Authors:** Samia Karkoutly, Yoshinori Takeuchi, Zahra Mehrazad Saber, Duhan Tao, Tsolmon Mendsaikhan, Rika Saikawa, Yuichi Aita, Yuki Murayama, Akito Shikama, Yukari Masuda, Naoya Yahagi

**Affiliations:** 1 Division of Endocrinology and Metabolism, Department of Medicine, Jichi Medical University, Shimotsuke, Tochigi 329-0498, Japan; 2 Nutrigenomics Research Group, Institute of Medicine, University of Tsukuba, Ibaraki 305-8575, Japan

**Keywords:** amino acids, urea cycle, transcription factor, fasting, *in vivo* imaging, nutrigenomics

## Abstract

Amino acid catabolism during fasting requires coordinated nitrogen disposal and glucose production, but the transcriptional logic linking the urea cycle to gluconeogenesis remains unclear. Forkhead box O (FoxO) transcription factors regulate fasting metabolism, and here we identify FoxOs as direct hepatic regulators of argininosuccinate synthase 1 (*Ass1*). Acute hepatic FoxO1/3a knockdown in fasted mice reduced Ass1 expression, lowered blood glucose, and altered urea-cycle amino acids, with decreased arginine and increased ornithine, even in *Klf15*-deficient livers. *Ass1* silencing phenocopied these effects and impaired glucose production from pyruvate and lactate, whereas Ass1 overexpression restored glucose production in FoxO-inhibited hepatocytes. Mechanistically, we identified a functional FoxO-binding element within an upstream Ass1 enhancer and confirmed its fasting-inducible activity by reporter assays, electrophoretic mobility shift assays, *in vivo* imaging, and chromatin immunoprecipitation. These findings establish a KLF15-independent FoxO-Ass1 axis that coordinates ureagenesis with gluconeogenesis during fasting and supports hepatic metabolic adaptation to nutrient deprivation.

## Highlights

• FoxOs directly activate hepatic Ass1 independently of KLF15
• A fasting-inducible FoxO-binding enhancer drives Ass1 expression *in vivo*
• FoxO or Ass1 knockdown lowers fasting blood glucose by impairing gluconeogenesis
• The FoxO-Ass1 axis coordinates the urea cycle with gluconeogenesis

## Graphical abstract

**Figure:**
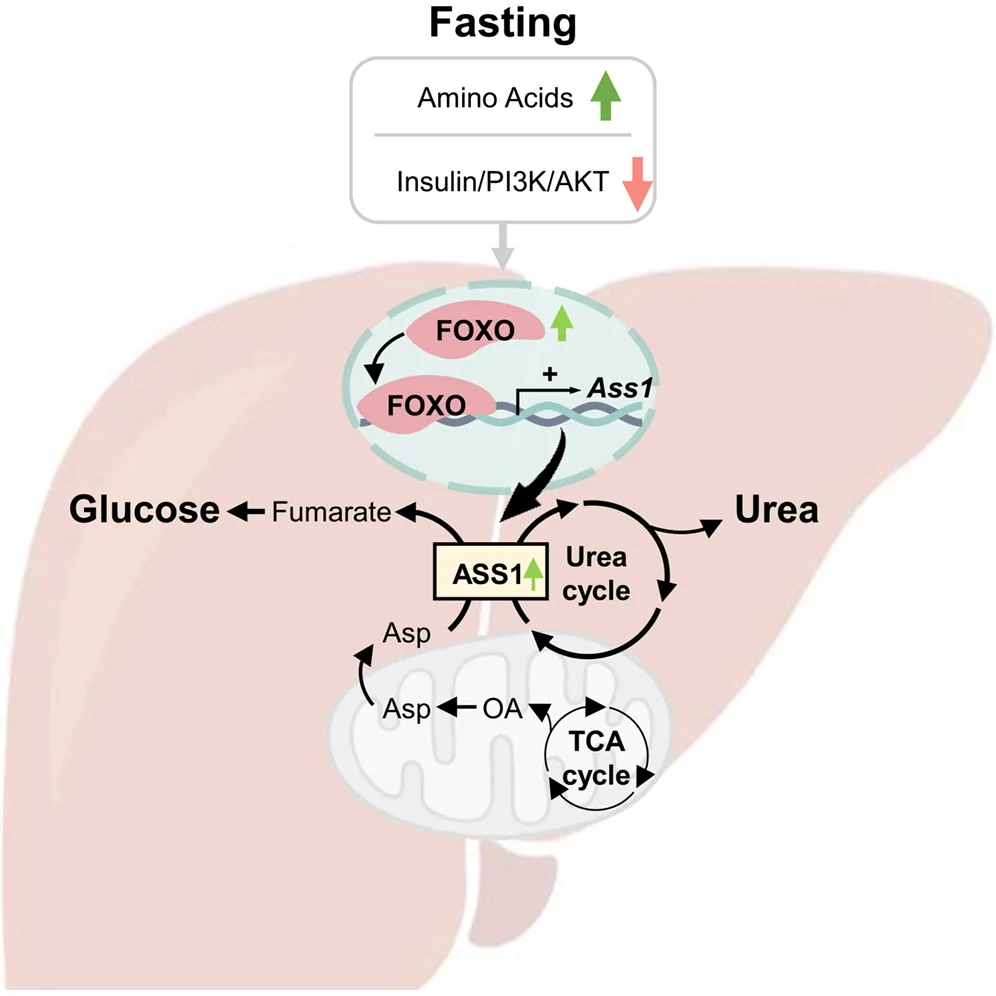

## Teaser

Molecular biology; Molecular mechanism of gene regulation; Endocrinology

## Introduction

Far beyond their role as proteinogenic molecules, amino acids serve as pivotal agents in metabolic integration,^1^^,^^2^^,^^3^ with their catabolic byproducts significantly influencing lipid and carbohydrate metabolism.^4^ Given this essential function, amino acid breakdown is subjected to strict regulatory control.^5^^,^^6^ Acting as the central orchestrator of metabolic integration, the liver dynamically processes amino acids, converting them into corresponding metabolites in response to the organism’s metabolic state and cellular demands.^5^^,^^7^

The deamination of amino acids generates nitrogenous waste in the form of free ammonia (NH_3_), a toxic byproduct that poses a metabolic burden on the organism.^4^^,^^8^ To prevent nitrogen accumulation and maintain homeostasis, this process is tightly linked to the urea cycle, which efficiently detoxifies NH_3_ by converting it into urea for excretion.^9^^,^^10^^,^^11^^,^^12^^,^^13^ Also known as the ornithine cycle, the urea cycle is a liver-specific pathway that comprises five core enzymes: carbamoyl-phosphate synthetase 1 (CPS1), ornithine transcarbamylase (OTC), argininosuccinate synthetase (ASS1), argininosuccinate lyase (ASL), and arginase 1 (ARG1).^14^ Notably, ASS1 and ASL, though classically defined as urea cycle enzymes, also serve a gluconeogenic function through fumarate production. This dual role is often overlooked in assessments of gluconeogenic rate control, particularly in the segment between pyruvate and phosphoenolpyruvate.^8^ Like other enzymes involved in amino acid catabolism, the expression of urea cycle genes is upregulated in response to increased substrate availability, such as during starvation or high dietary protein intake, thereby supporting enhanced protein degradation.^15^^,^^16^^,^^17^^,^^18^^,^^19^^,^^20^ It is worth noting that the enzymatic activities of urea cycle enzymes correlate with their mRNA levels, suggesting that their regulation occurs predominantly at the pre-translational level.^21^

Starvation represents a critical challenge that requires tight metabolic adaptation.^7^ During starvation, enhanced proteolysis supplies amino acids whose carbon skeletons are redirected toward gluconeogenesis.^22^^,^^23^^,^^24^ When amino acids are required for energy, their catabolism is initiated by the removal of the α-amino group via transamination and oxidative deamination.^4^^,^^5^ The resulting carbon skeletons then enter central metabolic pathways, either being oxidized through the tricarboxylic acid (TCA) cycle to generate ATP or funneled into gluconeogenesis for glucose synthesis.^7^^,^^25^ This reliance on gluconeogenesis brings it into several functional intersections with the urea cycle. First, upregulation of the urea cycle facilitates amino acid-based gluconeogenesis by removing amino groups from amino acids, thereby freeing their carbon skeletons for catabolic use.^23^^,^^24^ In addition, fumarate, which is produced from aspartate via the sequential actions of ASS1 and ASL within the urea cycle, plays a pivotal role in glucose synthesis, as it is converted to malate and subsequently oxidized to oxaloacetate, a key gluconeogenic precursor.^7^^,^^8^^,^^10^^,^^24^^,^^26^

Over a century ago, Graham Lusk demonstrated the influence of amino acids on glucose metabolism through the D/N ratio,^27^ highlighting the long-standing interest in nutrient-driven metabolic control. Despite this, the mechanisms that coordinate nitrogen disposal with glucose production, particularly the transcriptional regulation of urea cycle enzymes, remain underexplored. This gap persists even though the urea cycle was characterized as early as 1932 by Krebs et al.,^28^ several years before the discovery of the TCA cycle in 1937,^29^ whose transcriptional control is far better understood. Thus far, OTC remains the only urea cycle enzyme known to be directly regulated at the transcriptional level by Kruppel-like factor 15 (KLF15),^30^ and the wider regulatory network responsible for coordinated urea cycle induction during nutrient deprivation has yet to be elucidated.

KLF15, highly expressed in the liver and kidneys,^31^^,^^32^ regulates circadian nitrogen metabolism^33^^,^^34^ and directly influences catabolism of 11 of the 20 standard amino acids under high-protein dietary conditions, underscoring its broad role in amino acid homeostasis.^35^ Beyond amino acid metabolism,^30^ KLF15 also regulates lipid metabolism^36^ and glucose metabolism,^37^ integrating multiple nutrient-sensing signals to coordinate hepatic metabolic responses.

In line with the integrating role of KLF15, our previous work showed that the hepatic forkhead box O (FoxO)-KLF15 axis serves as a key conductor in the metabolic network directing the flow of macronutrients between starvation and overnutrition by linking amino acid-driven gluconeogenesis and glucose-driven lipogenesis under the influence of insulin.^38^ FoxO transcription factors, well-recognized for their conserved role in promoting health span and longevity across diverse species,^39^^,^^40^ play a crucial role in coordinating energy metabolism.^41^ They regulate glucose homeostasis by stimulating both gluconeogenesis and glycogenolysis,^42^^,^^43^^,^^44^^,^^45^ while also modulating lipid metabolism through upregulation of genes involved in fatty acid oxidation^46^ and downregulation of lipogenic genes.^47^ This multifaceted control positions FoxO transcription factors as key metabolic regulators bridging nutrient sensing and adaptive responses. Yet regulatory mechanisms are more complex. In response to high-protein nutritional states, we recently identified hepatic *Ass1* as a KLF15-independent gene under the direct control of FoxO transcription factors, highlighting an additional layer of transcriptional regulation in amino acid metabolism that operates beyond the KLF15 axis.^48^

In this study, we investigated the role of FoxO transcription factors in coordinating amino acid catabolism to glucose production during starvation. Specifically, we focused on the regulation of *Ass1*, a key urea cycle gene, as a direct FoxO target, and explored how this regulatory interaction may functionally link ureagenesis and gluconeogenesis. By clarifying this connection, we aim to uncover a transcriptional mechanism by which the liver aligns nitrogen disposal with energy production during starvation.

## Results

### FoxO transcription factors regulate the metabolism of amino acids involved in the urea cycle independently of KLF15 in the liver during fasting

**Figure 1:**
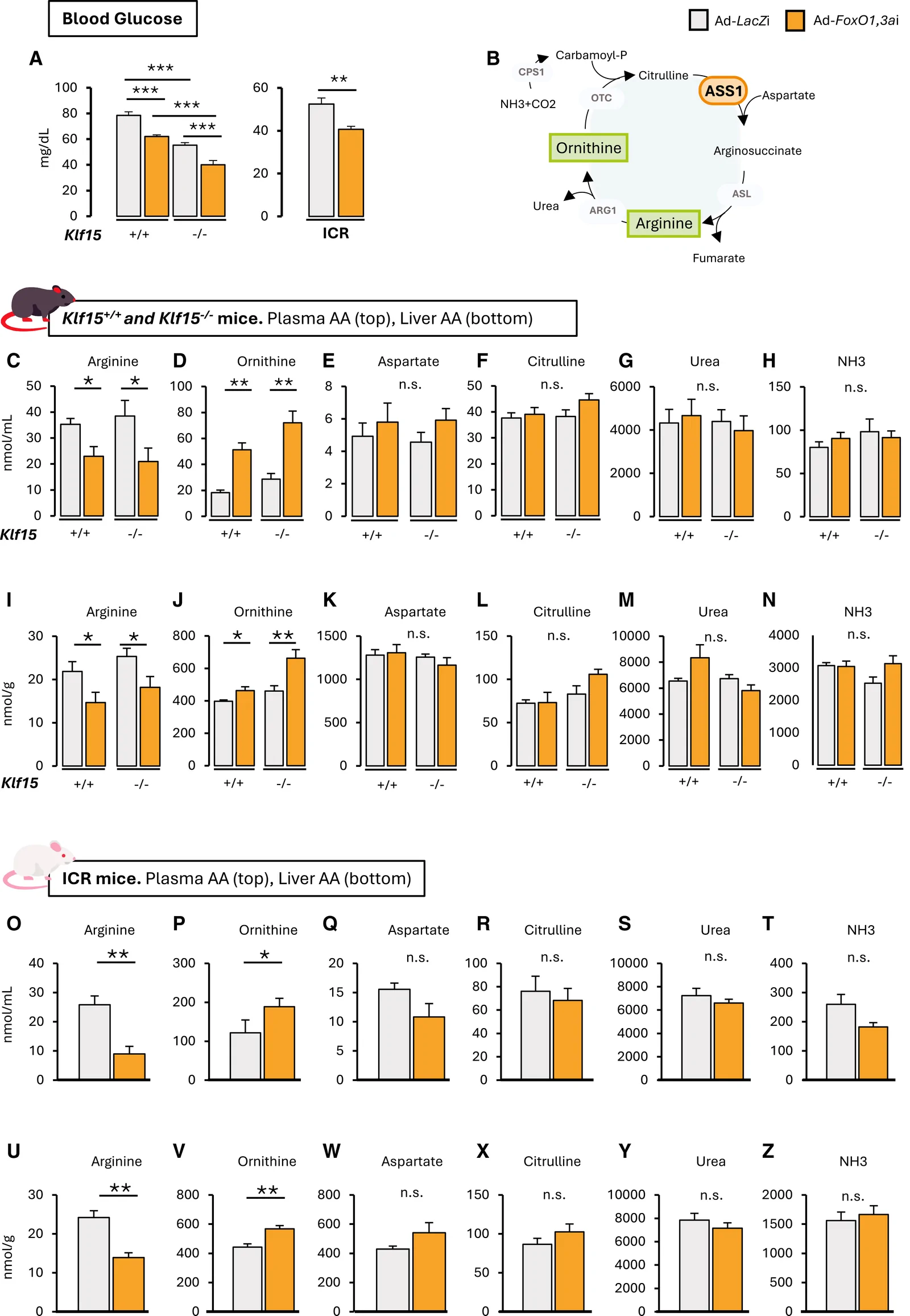
FoxO transcription factors regulate the metabolism of amino acids involved in the urea cycle independently of KLF15 in the liver during fasting (A) Left: blood glucose levels in *Klf15* wild-type and knockout C57BL/6J mice with hepatic *FoxO1* and *FoxO3a* knockdown (*n* = 8 per group). Right: blood glucose levels in ICR mice with hepatic *FoxO1* and *FoxO3a* knockdown (*n* = 12 per group). (B) Schematic representation of urea cycle enzymes and related amino acids. (C–H) Plasma levels of urea cycle-related amino acids, urea, and ammonia (NH_3_) in *Klf15* wild-type and knockout C57BL/6J mice with hepatic *FoxO1* and *FoxO3a* knockdown (*n* = 9–12 per group). (I–N) Hepatic levels of urea cycle-related amino acids, urea, and NH_3_ in *Klf15* wild-type and knockout C57BL/6J mice with hepatic *FoxO1* and *FoxO3a* knockdown (*n* = 8 per group). (O–T) Plasma levels of urea cycle-related amino acids, urea, and NH_3_ in ICR mice with hepatic *FoxO1* and *FoxO3a* knockdown (*n* = 7 per group). (U–Z) Hepatic levels of urea cycle-related amino acids, urea, and NH_3_ in ICR mice with hepatic *FoxO1* and *FoxO3a* knockdown (*n* = 8 per group). Plasma and hepatic amino acid levels were measured in ICR mice to ensure reproducibility of results. *FoxO1* and *FoxO3a* knockdown was performed using adenovirus-mediated shRNA (Ad-*FoxO1*,*3a*i). All groups of mice were sacrificed at the same period in the light cycle and blood and liver samples were collected in the fasted state following 24-h fasting. All values are presented as the mean with error bars representing the SEM. For multiple group comparisons, statistical significance for glucose measurements was determined by one-way ANOVA followed by Tukey’s multiple comparisons test, and for amino acid measurements, two-way ANOVA followed by Šídák’s multiple comparisons test was used. For two-group comparisons, unpaired two-tailed Student’s *t* test was used. Differences were considered significant if *p* < 0.05 (∗*p* < 0.05, ∗∗*p* < 0.01, and ∗∗∗*p* < 0.001).

In earlier work we have successfully shown that during fasting hepatic FoxO transcription factors accelerate amino acid breakdown and suppress lipogenesis all through the positive regulation of *Klf15*.^38^ Nevertheless, the possibility that FoxO transcription factors directly regulate other amino acid metabolic pathways remains a question worthy of investigation. In order to elucidate the individual role of each transcription factor, we designed a four-group mouse experiment; wild-type (WT) and *Klf15* knockout mice each treated with adenovirus-mediated *LacZ* small hairpin RNA (shRNA) (Ad-*LacZ*i) as a control and *FoxO1* and *FoxO3a* shRNA (Ad-*FoxO1*,*3a*i), which specifically targets both *FoxO1* and *FoxO3a*.^38^ Blood glucose levels and plasma and liver amino acid profiles were compared across the four groups in the fasted state. *Klf15* knockout mice showed lower blood glucose levels compared to the *Klf15* WT mice, and similar results were observed in Institute for Cancer Research (ICR) mice with hepatic *FoxO1* and *FoxO3a* knockdown (Figure 1A). Additionally, *FoxO* knockdown groups in both WT and *Klf15* knockout mice showed a significant decrease in blood glucose levels, with the *Klf15* knockout-*FoxO* knockdown group exhibiting the lowest fasting blood glucose levels of all, likely due to the combined effect of reduced *Klf15* and *FoxO* expressions. These data are consistent with previous reports.^30^^,^^49^ Regarding amino acid profile, two of the amino acids related to the urea cycle (Figure 1B) were notably affected. Specifically, plasma arginine levels (Figure 1C) were significantly decreased with *FoxO* knockdown in both *Klf15* WT and knockout groups, while *Klf15* knockout had no effect on its levels. By contrast, plasma ornithine levels (Figure 1D) showed a significant increase in the same groups, with no significant effect by *Klf15* knockout. Other urea cycle-related amino acids and metabolites, aspartate (Figures 1E–1H), citrulline, urea, and NH_3_ showed no significant change caused by *FoxO* knockdown or *Klf15* knockout. The complete plasma amino acid profile is shown in Figure S1. Branched chain amino acids (BCAAs) leucine, isoleucine and valine (Figures S1A–S1C), isoleucine and valine, along with tyrosine, and proline (Figures S1D and S1E) showed significant increase in their plasma levels due to *Klf15* knockout, whereas glutamine and glycine (Figures S1F and S1G) showed a significant decrease. These data are consistent with previous reports.^30^^,^^33^ It is worth mentioning that serine (Figures S1I and S1J) and threonine showed increased plasma levels with *FoxO* knockdown in both *Klf15* WT and knockout groups. To assess whether these metabolic changes are concordant across compartments, we measured hepatic amino acid levels in the previously analyzed mice. As shown in Figures 1I–1N, urea cycle-related metabolite levels were consistent between the liver and plasma under our experimental conditions. The unchanged plasma levels of urea are not indicative of maintained nitrogen homeostasis in plasma but rather reflect the presence of multiple feedback and compensatory regulatory loops that might control urea levels in many tissues.^50^ To ensure reproducibility of results, we analyzed both plasma and hepatic amino acids in ICR mice with *FoxO* knockdown in the fasted state. As Figures 1O, 1U, 1P and 1V show, and consistent with the previous results, arginine levels were significantly decreased, and ornithine levels were significantly increased in both plasma and liver. On the other hand, plasma and hepatic levels of aspartate (Figures 1Q and 1W), citrulline (Figures 1R and 1X), urea (Figures 1S and 1Y), and NH_3_ (Figures 1T and 1Z) showed no significant difference with *FoxO* knockdown. Additional data on the full hepatic amino acid profile during fasting, including comparisons with the *ad libitum*-fed state, are provided in Figure S2.

### Hepatic FoxO transcription factors control the urea cycle through *Ass1* during fasting

**Figure 2:**
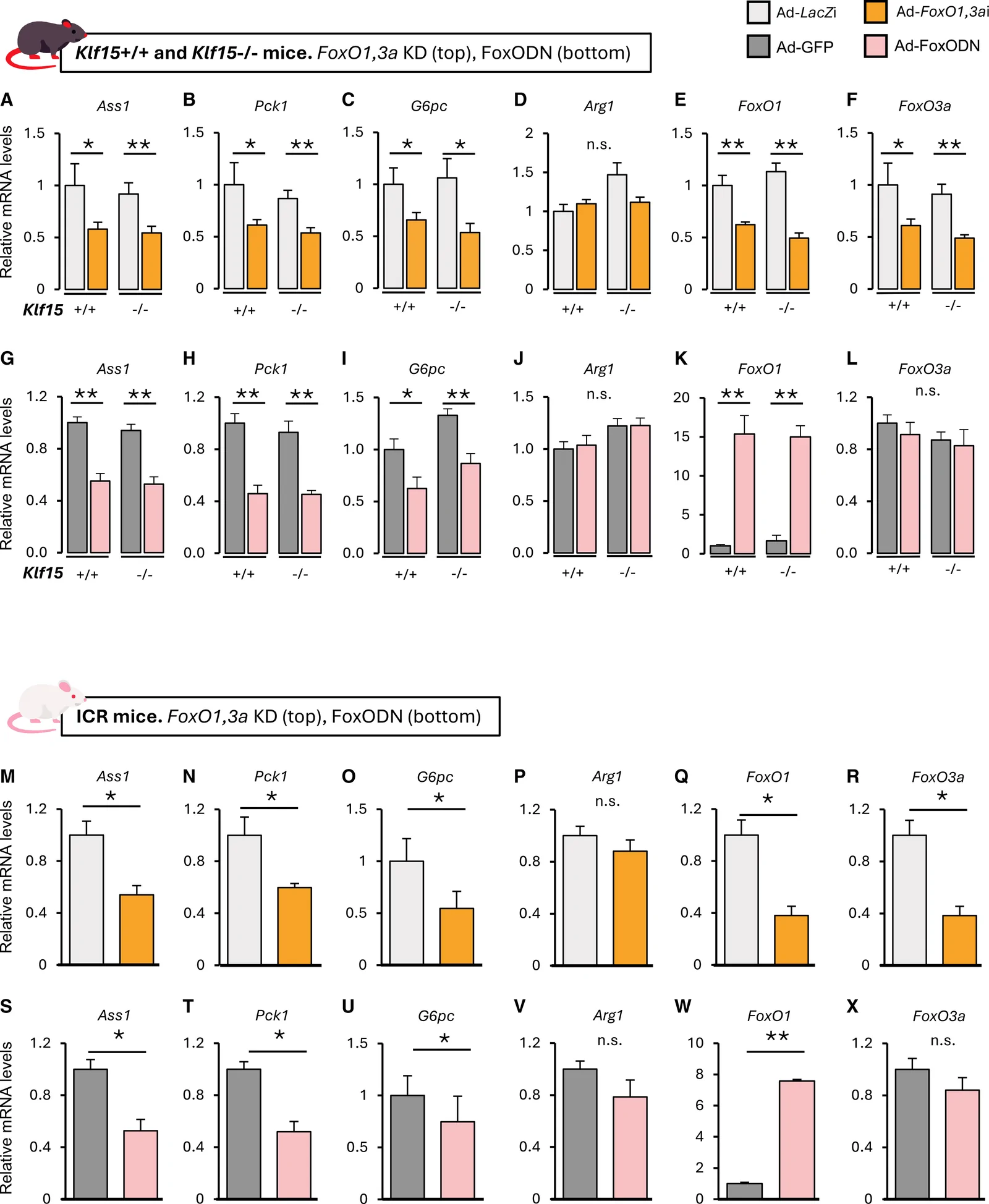
Hepatic FoxO transcription factors control the urea cycle through *Ass1* during fasting RT-qPCR analysis of liver RNA samples. (A–F) Relative gene expression in *Klf15* wild-type and knockout C57BL/6J mice with hepatic *FoxO1* and *FoxO3a* knockdown (*n* = 7–10 per group). (G–L) Relative gene expression in *Klf15* wild-type and knockout C57BL/6J mice with FoxO1 dominant negative overexpression (*n* = 6–7 per group). (M–R) Relative gene expression in ICR mice with hepatic *FoxO1* and *FoxO3a* knockdown (*n* = 8 per group). (S–X) Relative gene expression in ICR mice with FoxO1 dominant negative overexpression (*n* = 7 per group). Relative gene expression was analyzed in ICR mice with *FoxO1*,*3a* knockdown to ensure reproducibility of results. Relative gene expression was analyzed with FoxO1DN to exclude the possibility of artificial effects. As the correction of the gene expression level for each sample Cyclophilin A was used in (A–F) and (M–R). *Gapdh* was used in (G–L) and (S–X). *FoxO1* and *FoxO3a* knockdown was performed using adenovirus-mediated shRNA (Ad-*FoxO1*,*3a*i). FoxO1 dominant negative protein overexpression was performed using protein-expressing adenovirus (Ad-FoxODN). All groups of mice were sacrificed at the same period in the light cycle and liver samples were collected in the fasted state following 24-h fasting. All values are presented as the mean with error bars representing the SEM. For multiple group comparisons, statistical significance was determined by two-way ANOVA followed by Šídák’s multiple comparisons. For two-group comparisons statistical significance was determined by unpaired two-tailed Student’s *t* test. Differences were considered significant if *p* < 0.05 (∗*p* < 0.05, ∗∗*p* < 0.01, and ∗∗∗*p* < 0.001).

To have a better understanding of the specific contribution of FoxO transcription factors-mediated amino acid metabolism related to the urea cycle, and building on our previous findings that FoxO transcription factors regulate argininosuccinate synthase 1 gene (*Ass1*) under high-protein diet conditions,^48^ we evaluated its gene expression levels in *Klf15* WT and knockout mice treated with both Ad-*LacZ*i and Ad-*FoxO1*,*3a*i during fasting. As shown in Figure 2A, and in line with the previously shown amino acids plasma and hepatic levels, *Ass1* gene expression was significantly decreased with *FoxO* knockdown independently of *Klf15* knockout. *Pck1* (Figure 2B) and *G6pc* (Figure 2C), known targets of FoxO transcription factors, also showed decreased gene expression with *FoxO* knockdown again, with no difference between *Klf15* WT and knockout groups. *Arg1* (Figure 2D) expression showed no change across *FoxO* knockdown and *Klf15* knockout groups, and its expression level was used as a negative control. To further validate these findings in *Klf15* WT and knockout mice, we assessed gene expression levels using the adenovirus-mediated overexpression of dominant-negative FoxO1 (Ad-FoxODN). As shown in Figures 2G–2I, *Ass1*, *Pck1*, and *G6pc* gene expression showed a significant decrease with FoxO dominant-negative overexpression independently of *Klf15* knockout, while *Arg1* expression remained unchanged (Figure 2J), exhibiting essentially the same pattern as the *FoxO* knockdown data and further confirming that this regulation operates independently of the KLF15 axis. Figure 2K shows FoxO1 protein overexpression using Ad-FoxODN. The gene expression levels were also assessed in ICR mice treated with both Ad-*LacZ*i and Ad-*FoxO1*,*3a*i to ensure reproducibility of results. In accordance with the abovementioned data, *Ass1*, *Pck1* and *G6pc* gene expression showed a significant decrease with lower *FoxO* gene expression (Figures 2G–2I). The adenovirus-mediated overexpression of dominant-negative FoxO1 (Ad-FoxODN)^38^ exhibited essentially the same effects on expression levels of these genes to exclude the possibility of artificial effects (Figures 2M–2R). Figures 2E, 2F, 2K, and 2L show the knockdown efficiency of both *FoxO1* and *FoxO3a* using Ad-*FoxO1*,*3a*i, and Figure 2Q shows FoxO1 protein overexpression using Ad-FoxODN. Additional comparisons of gluconeogenic and urea-cycle gene expression, together with ASS1 protein levels in total liver lysates from fasted and *ad libitum*-fed mice, are shown in Figure S3.

### Metabolic phenotypes analogous to *FoxO* knockdown were observed following *Ass1* knockdown during fasting

**Figure 3:**
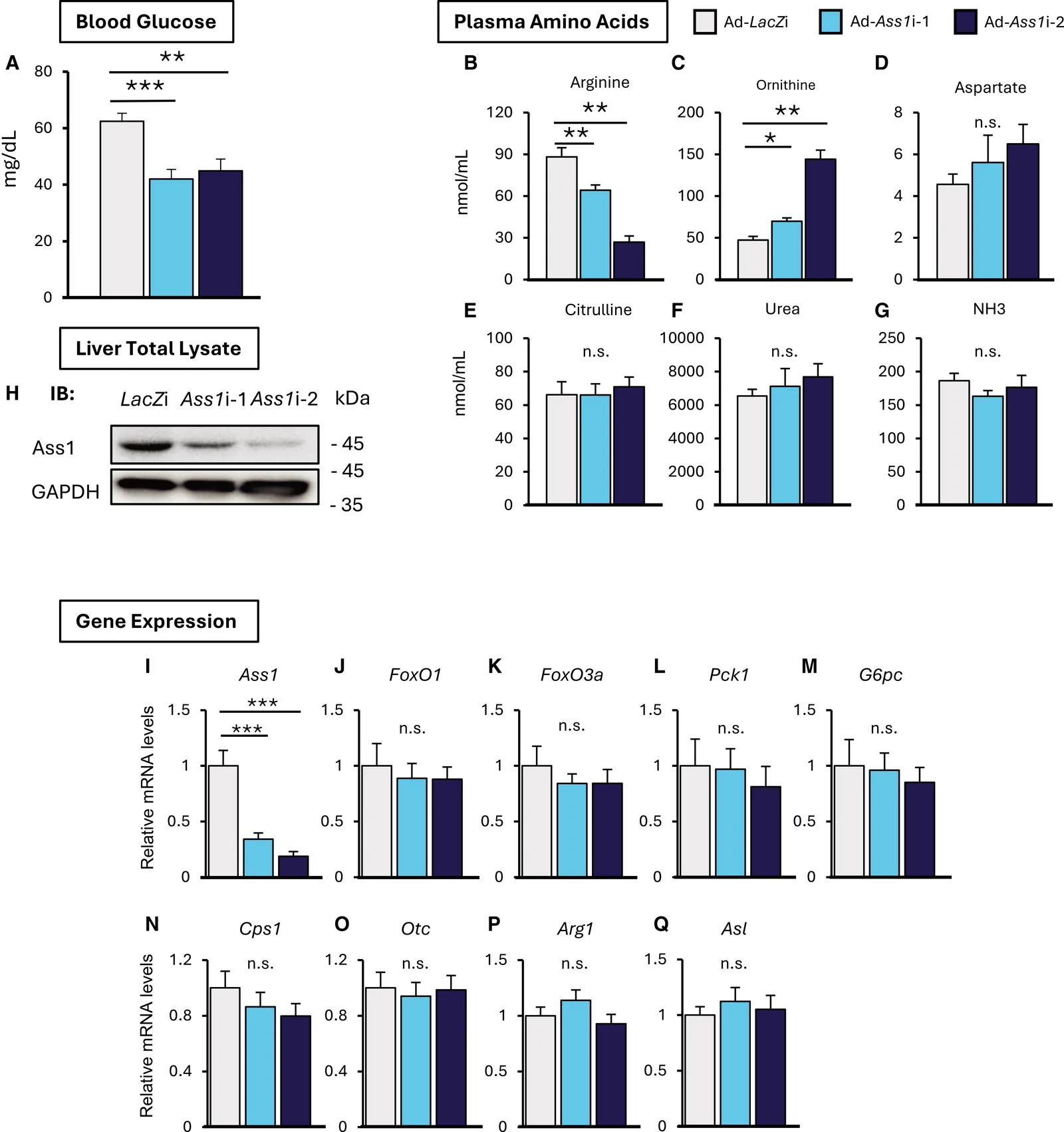
Metabolic phenotypes analogous to *FoxO* knockdown were observed following *Ass1* knockdown during fasting (A) Blood glucose levels in ICR mice with hepatic *Ass1* knockdown (*n* = 12 per group). (B–G) Plasma levels of urea cycle-related amino acids, urea, and ammonia (NH_3_) in ICR mice with hepatic *Ass1* knockdown (*n* = 8 per group). (H) Immunoblot analysis of ASS1 protein using liver total lysate from mice in the fasted state (samples were pooled from 3 mice). GAPDH was detected as internal loading control. Other internal loading controls were also detected in Figure S4. (I–Q) Relative gene expression in ICR mice with hepatic *Ass1* knockdown (*n* = 8 per group). *Ass1* knockdown was performed using adenovirus-mediated shRNA (Ad-*Ass1*i-1, Ad-*Ass1*i-2). As the correction of the gene expression level for each sample Cyclophilin A was used. All groups of mice were sacrificed at the same period in the light cycle and blood and liver samples were collected in the fasted state following 24-h fasting. All values are presented as the mean with error bars representing the SEM. For multiple-group comparisons, statistical significance was determined by one-way ANOVA followed by Dunnett’s multiple comparisons test. Differences were considered significant if *p* < 0.05 (∗*p* < 0.05, ∗∗*p* < 0.01, and ∗∗∗*p* < 0.001).

Hepatic *Ass1* was silenced with two adenoviral shRNA constructs (*Ass1*i-1 and *Ass1*i-2)^48^ to assess its potential role in mediating the regulation of urea cycle-related amino acid metabolism by FoxO transcription factors. Blood glucose levels and plasma and liver amino acid profiles were compared across the groups in the fasted state. Both *Ass1* knockdown groups showed lower blood glucose levels compared to the Ad-*LacZ*i group (Figure 3A), which mirrors the phenotype caused by *FoxO* knockdown (Figure 1A). Amino acid measurement in the plasma of ICR mice during fasting revealed that *Ass1* knockdown phenocopied the effect of *FoxO* knockdown (Figures 3B–3G). Arginine levels were decreased and ornithine levels were increased, whereas aspartate, citrulline, urea, and NH_3_ (Figures 3B–3G) levels showed no significant differences. Upon analyzing total liver lysate of ICR mice with *Ass1* knockdown (Figure 3H), we detected reduction in ASS1 protein levels in *Ass1*i-1 and *Ass1*i-2 treated groups during fasting. *Ass1* shRNA induced the knockdown of endogenous *Ass1* to levels comparable with the downregulation of *Ass1* upon *FoxO* knockdown (Figure 3I). At the same time, *FoxO1*, *FoxO3a*, and other urea cycle genes showed no differences in *Ass1*i groups (Figures 3J–3Q). The two shRNAs exerted essentially the same effects on amino acid levels and the expression levels of other urea cycle genes, excluding the possibility of off-target effects. Collectively, our findings suggest that *Ass1* is a critical direct target of the FoxO transcription factors in the regulation of the urea cycle-related amino acid metabolism during fasting.

### Hepatic FoxO signaling and ASS1 are both required for gluconeogenesis from lactate and pyruvate *in vivo*

**Figure 4:**
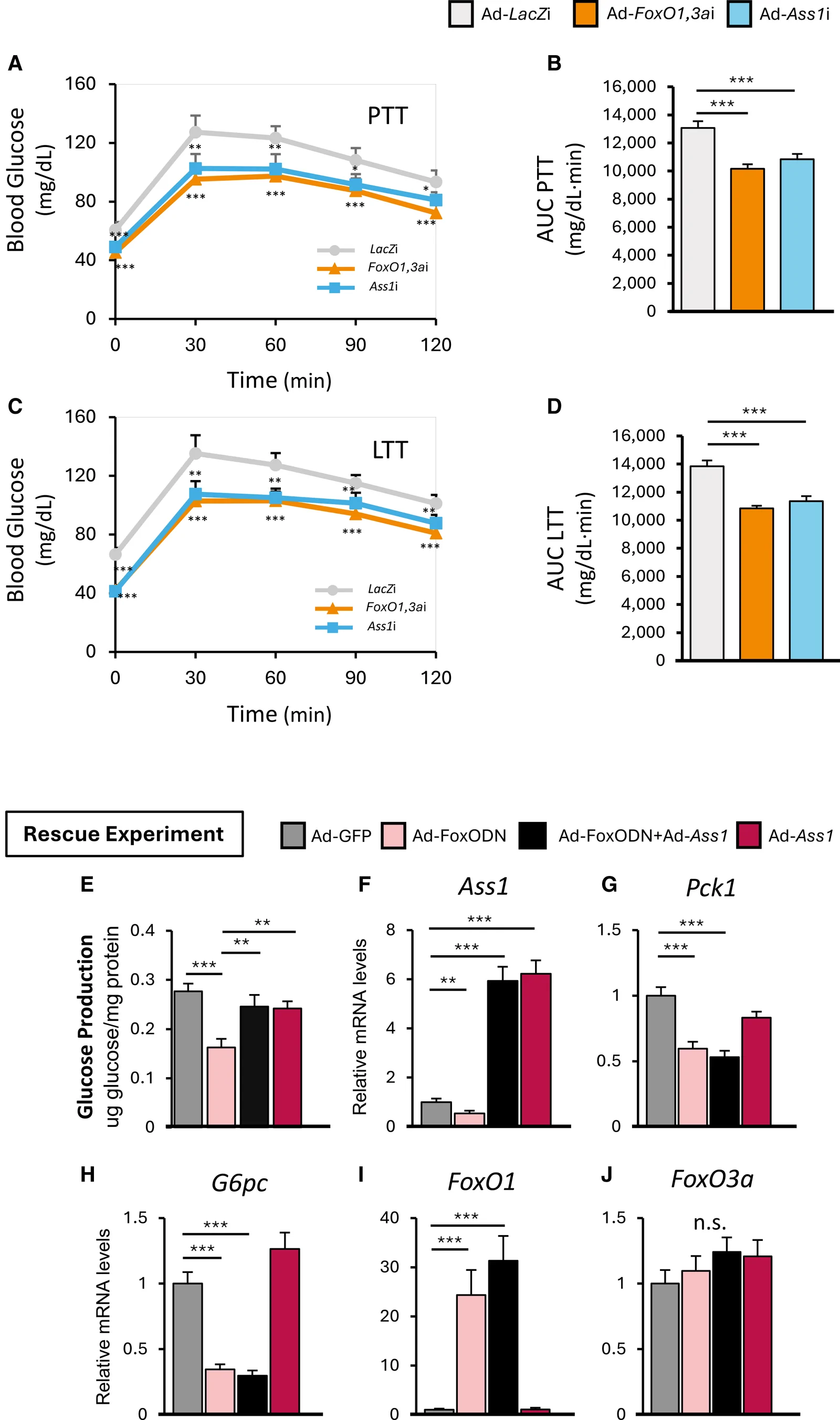
ASS1 effect on glucose production from its substrates both *in vivo* and in primary hepatocytes (A and C) Blood glucose levels over time during pyruvate tolerance test (PTT) and lactate tolerance test (LTT), respectively, in ICR mice with hepatic *FoxO1* and *FoxO3a* knockdown or *Ass1* knockdown. Male ICR mice were injected with Ad-*LacZ*i, Ad-*FoxO*i, or Ad-*Ass1*i adenoviruses for 5 days, fasted for 24 h, and then intraperitoneally injected with pyruvate (2 g/kg) or lactate (2 g/kg), with blood glucose levels measured at the indicated time points (*n* = 14 per group). (B and D) Area under the curve (AUC; mg/dL·min) of blood glucose levels during PTT and LTT, respectively (*n* = 14 per group). (E) Glucose production measured in the culture medium of primary hepatocytes isolated from ICR mice and transduced with adenoviruses expressing GFP as control, FoxO dominant-negative (Ad-FoxODN) 10 m.o.i., *Ass1* (Ad-*Ass1*) 1 m.o.i., or Ad-FoxODN together with Ad-*Ass1* (Rescue). Glucose output was normalized to total protein content (*n* = 4 independent experiments). (F–J) RT-qPCR analysis of *Ass1* (F), *Pck1* (G), *G6pc* (H), *FoxO1* (I), and *FoxO3a* (J) mRNA levels in primary hepatocytes transduced as described in (E). Gene expression was normalized to Cyclophilin A and expressed relative to the GFP control group (*n* = 4 independent experiments). *FoxO1* and *FoxO3a* knockdown was performed using adenovirus-mediated shRNA (Ad-*FoxO1*,*3a*i). *Ass1* knockdown was performed using adenovirus mediated shRNA (Ad-*Ass1*i). All values are presented as the mean with error bars representing the SEM. For multiple-group comparisons, statistical significance for PTT and LTT time-course curves was determined by one-way ANOVA followed by Dunnett’s multiple comparisons test at each timepoint, and for PTT and LTT AUC by one-way ANOVA followed by Dunnett’s multiple comparisons test. For the rescue experiment, statistical significance for glucose measurements was determined by one-way ANOVA followed by Tukey’s multiple comparisons test, and for qPCR data by Welch’s *t* test with Šídák correction for multiple comparisons. Differences were considered significant if *p* < 0.05 (∗*p* < 0.05, ∗∗*p* < 0.01, and ∗∗∗*p* < 0.001).

To directly assess whether the hypoglycemia observed following *FoxO* and *Ass1* knockdown reflects impaired gluconeogenic capacity, we performed pyruvate tolerance tests (PTTs) and lactate tolerance tests (LTTs) in mice following adenovirus-mediated hepatic knockdown of *FoxO*1 and *FoxO3a* (Ad-*FoxO*i) or *Ass1* (Ad-*Ass1*i), using Ad-*LacZ*i-injected mice as controls. Both *FoxO*i and *Ass1*i mice displayed reduced blood glucose production following intraperitoneal pyruvate challenge compared to controls (Figures 4A and 4B). Consistent with these findings, LTT revealed that conversion of lactate to glucose was similarly impaired in both knockdown groups (Figures 4C and 4D). These results demonstrate that both FoxO and ASS1 are required for efficient hepatic gluconeogenesis from physiological carbon substrates *in vivo*.

### *Ass1* overexpression rescues the reduction in gluconeogenesis caused by FoxO inhibition in primary hepatocytes

To determine whether the reduced *Ass1* expression contributes to the suppression of glucose production caused by FoxO inhibition, we transduced primary hepatocytes with an adenovirus expressing *Ass1* in addition to Ad-FoxODN. While Ad-FoxODN alone significantly reduced glucose output compared to GFP controls, co-expression of *Ass1* restored glucose production to control levels (Figure 4E), indicating that ASS1 can compensate for the partial downregulation of genes *Pck1* and *G6pc* (Figures 4J and 4H). Figure 4F shows *Ass1* downregulation in the FoxODN group and overexpression in both *Ass1* and rescue groups. Similarly, Figure 4I shows FoxODN overexpression using Ad-FoxODN.

### Identification of FoxO transcription factors binding region upstream of *Ass1* gene

**Figure 5:**
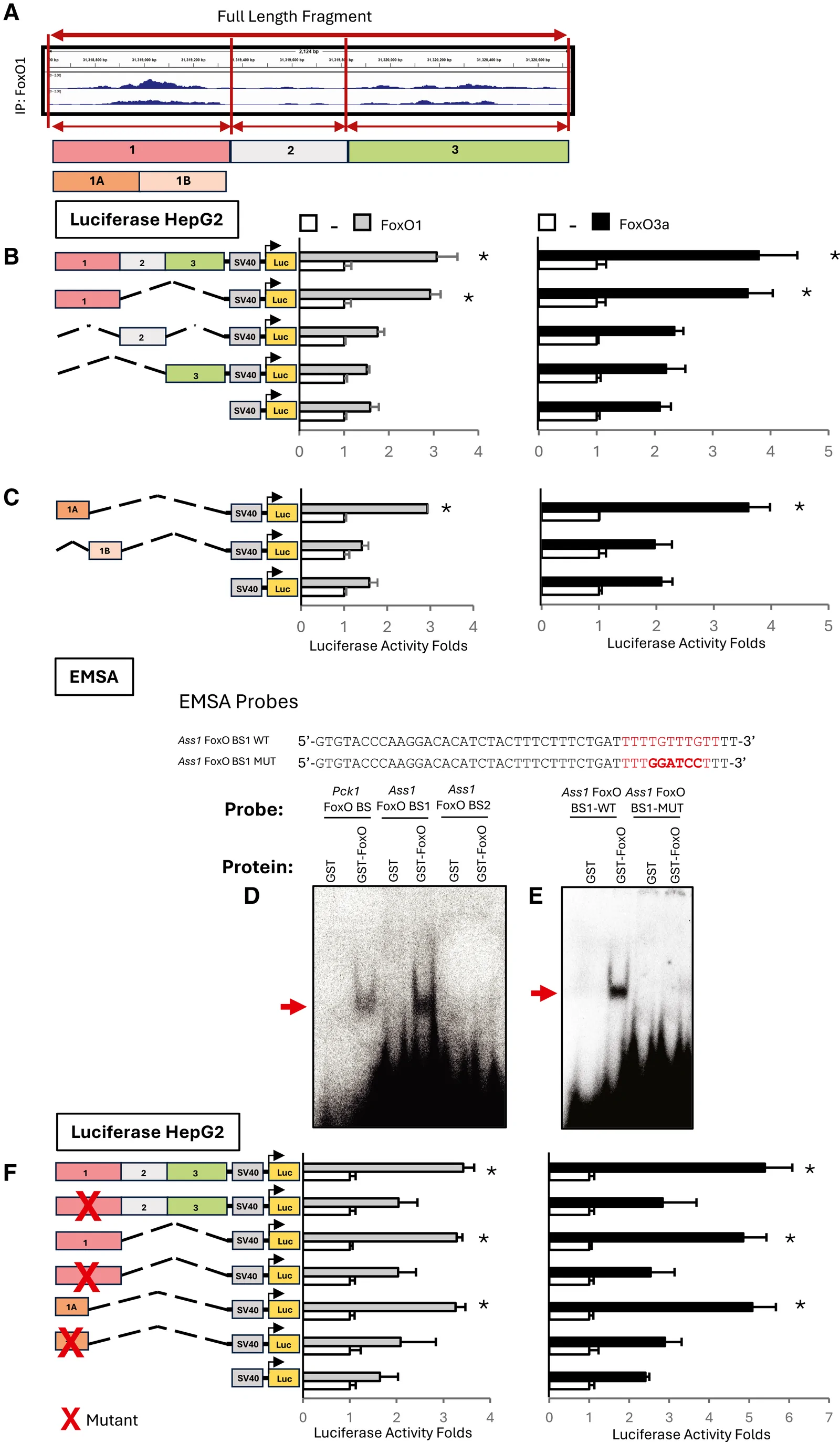
Identification of FoxO transcription factors binding region upstream of *Ass1* gene (A) Top: the results of ChIP-seq with anti-FoxO1 antibody in livers of unfed mice obtained from ChIP-Atlas (GSM3381273)^51^; bottom: simplified representation of the structure of the full fragment, fragment 1, fragment 2, fragment 3, and the subdivisions of fragment 1 i.e., small fragment 1A and small fragment 1B. (B and C) Enhancer analysis of FoxO1 and FoxO3a on *Ass1* enhancer and its fragments. *FoxO1* and *FoxO3a* expression plasmids were co-transfected with the indicated *Ass1* enhancer firefly luciferase reporter plasmids in HepG2 cells. (D and E) Electrophoretic mobility shift assay (EMSA) using radiolabeled probe for (D) the two JASPAR predicted FoxO binding sites (Figure S6) in the smallest fragment 1A from the *Ass1* enhancer and FoxO binding site in the promoter of *Pck1* as a positive control; (E) the wild-type (WT) and mutant (Mut) predicted FoxO binding site 1 incubated with GST and GST-FoxO recombinant proteins. BS, binding site; WT, wild-type; Mut, mutant. (F) Enhancer analysis of FoxO1 and FoxO3a on mutant *Ass1* enhancer and its fragments. *FoxO1* and *FoxO3a* expression plasmids were co-transfected with the indicated *Ass1* enhancer firefly luciferase reporter plasmids in HepG2 cells. All values are presented as the mean with error bars representing the SEM. Datasets were assessed by Student’s *t* test for unpaired samples. Differences were considered significant if *p* < 0.05 (∗*p* < 0.05).

To determine FoxO transcription factors binding sites (BSs) in the *Ass1* enhancer region (Figure S5), we performed enhancer analysis using various *Ass1*-enhancer-luc constructs in HepG2 cells. Possible FoxO transcription factors BSs in the enhancer region upstream of the *Ass1* gene have been identified using chromatin immunoprecipitation (ChIP)-Atlas (GSM3381273)^51^ (Figure 5A, top). In order to locate the BS, the mentioned area was subdivided into smaller fragments and cloned, along with the full-length fragment (Figure 5A, bottom), into luciferase reporter plasmid to be studied separately through evaluating the luciferase activity in HepG2 cells. As the data show, both full-length and fragment 1 (Figure 5B) and subdivision A of fragment 1 (Figure 5C) showed significant luciferase activity to both *FoxO1* and *FoxO3a* expression plasmids compared to other fragments and to the control.

To support these findings, an electrophoretic mobility shift assay (EMSA) was performed. For that, two radiolabeled probes for the two high score BSs in fragment 1A based on JASPAR analysis results were designed (Figures S6A–S6D) and checked against FoxO BS on *Pck1* promoter as a positive control. EMSA results showed that the recombinant FoxO protein binds to the DNA radiolabeled probe of BS number 1 (*Ass1* FoxO BS1) (Figure 5D). Then as a next step, we designed a probe for mutated *Ass1* FoxO BS1 and no band was observed (Figure 5F).

Consistent with these findings, when we mutated this predicted BS in the full-length fragment, fragment 1 and fragment 1A, the activation of the luciferase gene by both *FoxO1* and *FoxO3a* expression plasmids in HepG2 cells was completely abolished as shown in Figure 5F. Collectively, these findings identify the specific FoxO-BS essential for enhancer-mediated activation.

### The identified region is required for hepatic *Ass1* response to fasting

**Figure 6:**
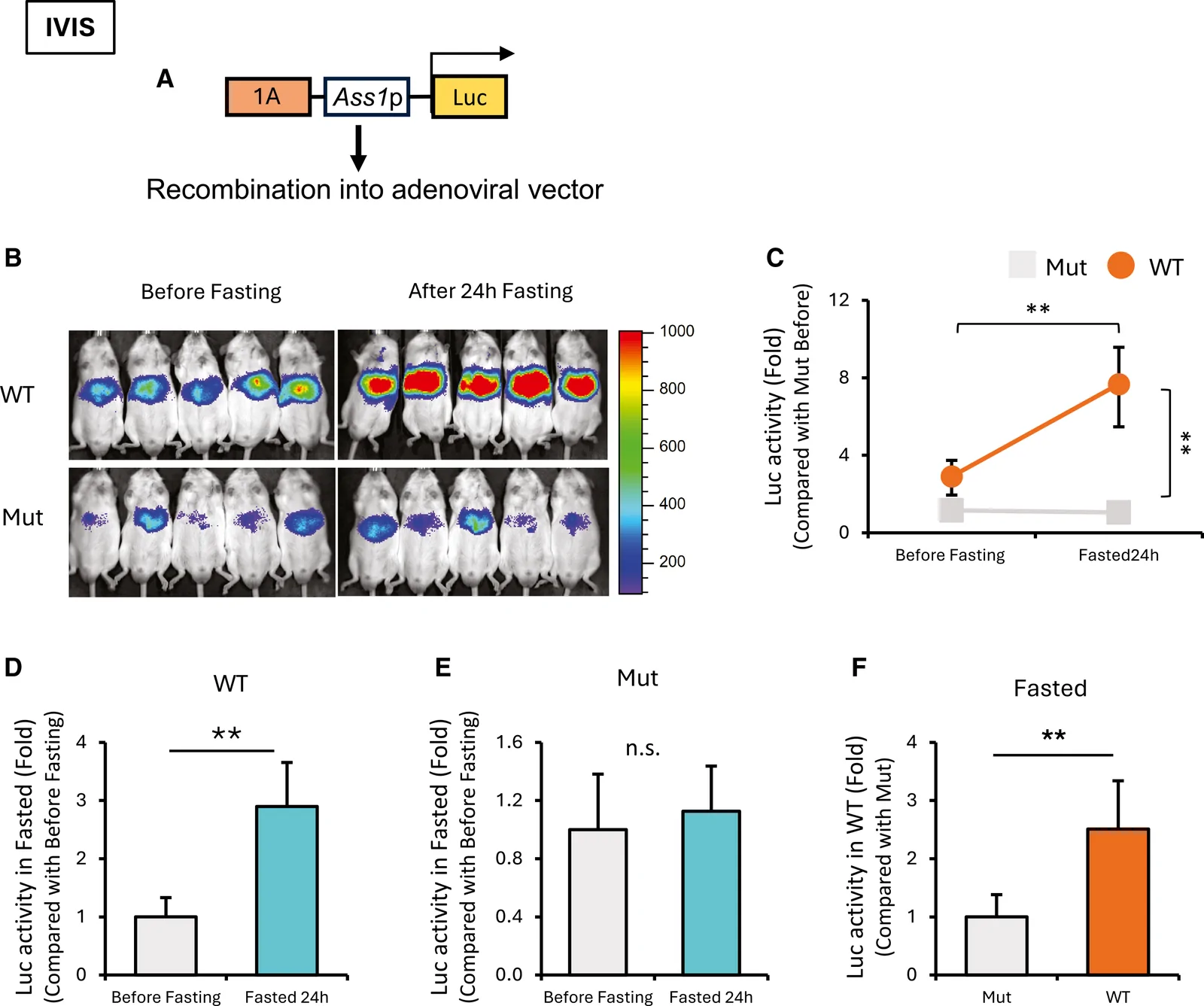
The identified region is required for hepatic *Ass1* response to fasting (A) The structure of the Ad-Luc showing the identified FoxO binding site in the enhancer region of *Ass1* linked to the Luc reporter using *Ass1* native promoter. (B–F) *In vivo* Ad-Luc enhancer analyses using Ad-FoxOBS-*Ass1*Promoter-Luc both wild-type and mutant. Representative images (B) and hepatic luciferase activities (C–F) of mice injected with Ad-FoxOBS-*Ass1*Promoter-Luc both wild-type and mutant are shown (*n* = 10 per group). (D) Enhancer activity in the liver for wild-type binding site after 24-h fasting is expressed relative to activity before fasting, to adjust for mouse-to-mouse differences in enhancer expression in Ad-treated mice. (E) Enhancer activity in the liver for mutant binding site after 24-h fasting is expressed relative to activity before fasting, to adjust for mouse-to-mouse differences in enhancer expression in Ad-treated mice. (F) Enhancer activity in the liver after 24-h fasting for wild-type binding site is expressed relative to activity of mutant binding site, to adjust for mouse-to-mouse differences in enhancer expression in Ad-treated mice. BS, binding site; WT, wild-type; Mut, mutant. Data were assessed using the paired two-tailed Student’s *t* test. Differences were considered significant if *p* < 0.05 (∗*p* < 0.05 and ∗∗*p* < 0.01). Error bars mean SEM.

To validate these findings, we assessed the effect of fasting on transcriptional activity *in vivo* by engineering the smallest fragment containing the identified FoxO BS (Figure 5) both the WT and the mutated versions into a luciferase reporter plasmid connected to *Ass1* native promoter (Figure 6A) and delivered adenovirally to the liver. The transcriptional activity was assessed by measuring luciferase activity with an In Vivo Imaging System (IVIS) imaging system^36^^,^^52^^,^^53^^,^^54^ before and after fasting. As shown in Figures 6B–6F, the reporter activity of the WT FoxO BS construct significantly increased in the fasted state compared to the fed state (Figures 6C and 6D) and compared to the mutated version (Figures 6C and 6E). The response of the mutated FoxO BS construct was significantly attenuated (Figures 6C, 6E, and 6F) suggesting that this site is necessary for the activation of *Ass1*.

### FoxO transcription factors bind to hepatic *Ass1* enhancer during fasting

**Figure 7:**
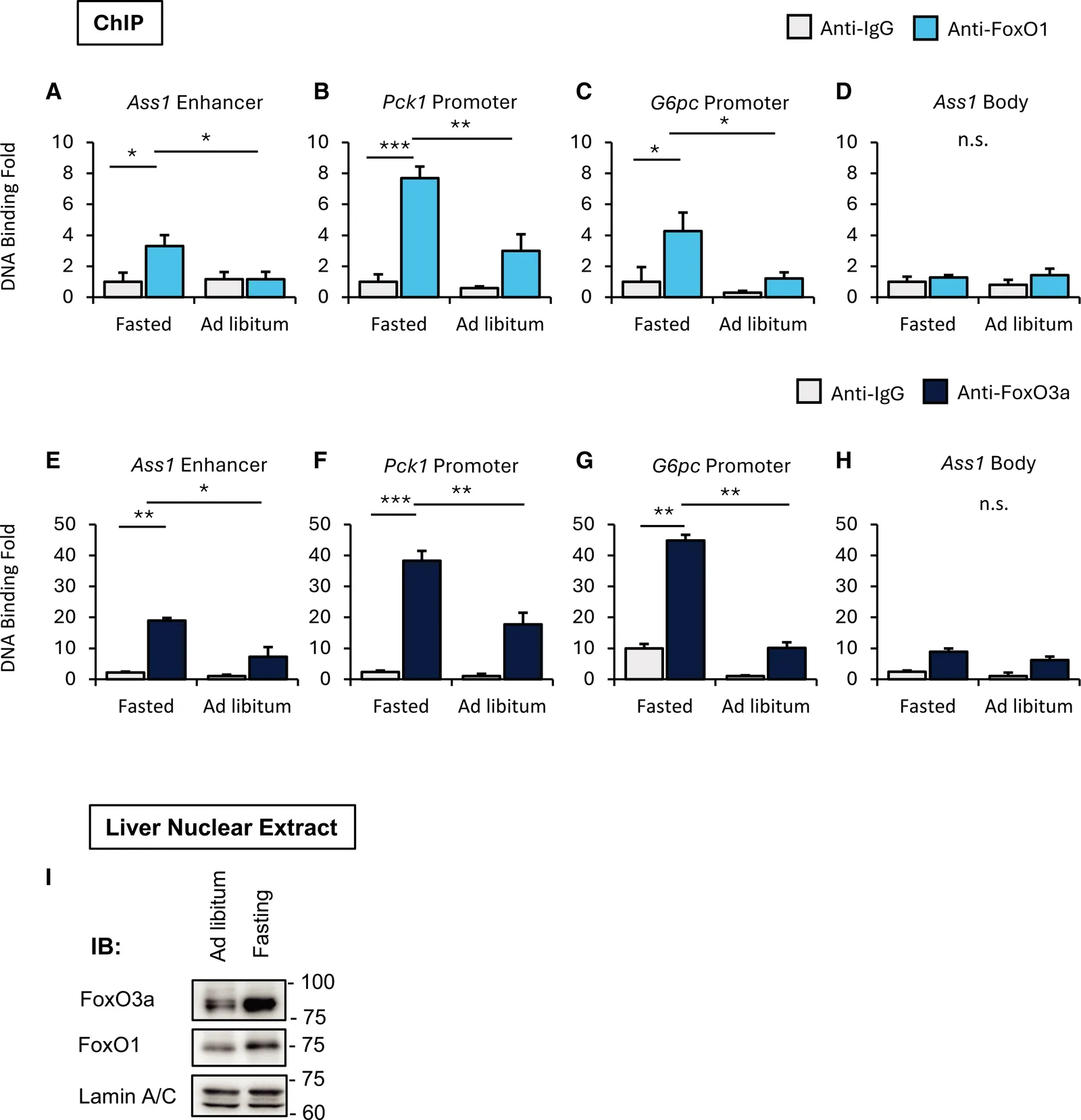
FoxO transcription factors bind to hepatic *Ass1* enhancer during fasting (A–D) Chromatin immunoprecipitation (ChIP) assay to determine the interaction of hepatic FoxO proteins with *Ass1* DNA enhancer region using anti-FoxO1 antibody and normal mouse anti-IgG antibody as a control (*n* = 6 per group). (E–H) ChIP assay to determine the interaction of hepatic FoxO proteins with *Ass1* DNA enhancer region using anti-FoxO3a antibody and normal mouse anti-IgG antibody as a control (*n* = 6 per group). (A and E) FoxO proteins binding to *Ass1* enhancer in liver detected using primer set designed around the identified FoxO binding site as shown in Figure 5. (B and F) *Pck1* promoter FoxO transcription factor binding site and (C and G) *G6pc* promoter FoxO transcription factor binding site used as a positive control. (D and H) Used as a negative control. (I) Immunoblot analysis of FoxO1 and FoxO3a proteins using liver nuclear extracts from mice in *ad libitum*-fed and fasted state (samples were pooled from 3 mice). Lamin A/C was detected as an internal loading control for nuclear protein. All groups of mice were sacrificed in the light cycle and liver samples were collected in the fasted state following 24-h fasting or *ad libitum*-fed as indicated. For multiple group comparisons, statistical significance was determined by two-way ANOVA followed by Šídák’s multiple comparisons test. Differences were considered significant if *p* < 0.05 (∗*p* < 0.05, ∗∗*p* < 0.01, and ∗∗∗*p* < 0.001). Error bars mean SEM.

To have a deeper insight into the regulatory mechanism of FoxO transcription factors over *Ass1* gene expression *in vivo*, we employed ChIP assay to evaluate the binding ability of FoxO transcription factors to *Ass1* enhancer during the fasted state compared to the *ad libitum*-fed state using both anti-FoxO1 and anti-FoxO3a antibodies against anti-IgG antibody. As Figures 7A–7E depicted, FoxO1 and FoxO3a binding occupancy to the identified FoxO BS on the *Ass1* enhancer region (Figure 5) was significantly increased during fasting compared with the *ad libitum*-fed state. *Pck1* (Figures 7B and 7F) and *G6pc* (Figures 7C and 7G) promoter FoxO transcription factor BS were used as a positive control and showed FoxO transcription factor occupancy on the corresponding promoter. *Ass1* gene body (Figures 7D and 7H) was used as negative control. To reduce potential higher background in our ChIP experiments, we performed CUT&Tag assays under fasting conditions in mice treated with both Ad-*LacZ*i and Ad-*FoxO1*,*3a*i as a direct genetic depletion control for antibody specificity Figure S7. As shown in Figure S7A, FoxO1 enrichment at the *Ass1* enhancer was significantly enriched in the *LacZ* control group and was markedly reduced upon *FoxO1/3a* knockdown. Significant enrichment at the *Pck1* promoter was similarly abolished by *FoxO1/3a* knockdown, while no significant enrichment was detected at the *Ass1* gene body. The complete elimination of enrichment upon *FoxO1/3a* knockdown reflects specific FoxO1 occupancy and is not attributable to non-specific binding. Next, upon examining hepatic nuclear FoxO1/FoxO3a protein levels (Figure 7I) we show how nuclear FoxO1 and FoxO3a protein levels increase during fasting compared to *ad libitum*-fed. Taken all together, these findings suggest that nuclear FoxO transcription factor availability during fasting directly stimulates *Ass1* transcription in the liver.

## Discussion

In this study, we identified a novel role for FoxO transcription factors as direct regulators of hepatic *Ass1*, a key urea cycle gene. This regulation occurs independently of KLF15 and orchestrates changes in urea cycle-related amino acid levels during starvation. By investigating this FoxO-*Ass1* interaction, we revealed a transcriptional mechanism that may functionally coordinate ureagenesis and gluconeogenesis, aligning nitrogen disposal with the energy production needs of the liver during nutrient deprivation.

Our group previously demonstrated that hepatic FoxO transcription factors control amino acid metabolism via the KLF15 pathway using a transcription factor expression library (TFEL) scan method,^38^^,^^55^ and subsequently confirmed that *Ass1* represents a direct FoxO target operating independently of KLF15.^48^

To adapt to fluctuations in caloric intake, organisms adjust the balance between carbohydrate and amino acid utilization as energy sources.^25^ When amino acids are catabolized to meet energy demands, the resulting amino nitrogen is primarily converted into urea and excreted, reflecting the body’s capacity to maintain nitrogen balance while utilizing the carbon skeletons for energy.^14^ The upregulation of the five urea cycle enzymes during starvation is well documented (Figures S3A and S3F–S3J).^17^ However, the transcriptional mechanisms that regulate this adaptive response have remained largely unresolved. To date, only *OTC* has been identified as a transcriptional target of KLF15,^30^ leaving the broader regulatory landscape governing urea cycle activation during nutrient deprivation still poorly understood.

Our data revealed that reduced availability of FoxO transcription factors significantly affected the plasma and hepatic levels of arginine and ornithine (Figure 1). Interestingly, the concentrations of citrulline and aspartate, the two direct substrates of ASS1, as well as NH_3_ and urea, remained largely unchanged in both the plasma and liver. Consistently, the expression of *Arg1*, which hydrolyzes arginine into urea and ornithine, also remained stable (Figure 2). This overall stability of the urea cycle, despite perturbations in individual components, can be attributed to the inherent robustness and adaptability of cellular metabolic networks. Such networks maintain homeostasis through redundant pathways, feedback regulation, and compensatory mechanisms that buffer against fluctuations in gene expression and environmental stressors.^56^ These characteristics, along with the nonlinear dynamics and the complexity of interactions among numerous metabolites, often obscure the identification of discrete regulatory nodes within metabolic circuits.^50^

During starvation, when amino acid catabolism is elevated, the urea cycle is pushed to operate at full capacity. Under these conditions, ASS1 functions as the rate-limiting step, with its activity primarily regulated by hormonal and nutritional cues.^57^ Glucocorticoids and glucagon are known to enhance ASS1 activity, while insulin suppresses it.^58^ Despite the central role of ASS1 in regulating ureagenesis, only a single transcription factor, Sp1, has been experimentally confirmed to bind its promoter region to date.^59^ In the present study, we identified FoxO transcription factors as novel direct regulators of hepatic *Ass1* expression during starvation, acting independently of KLF15 (Figure 2). We further demonstrated that FoxO-binding elements within the *Ass1* enhancer are essential for its transcriptional activation in response to nutrient deprivation (Figure 6).

There are two potential mechanisms by which FoxO binding to the *Ass1* enhancer may increase. One possibility is an increased amount of nuclear FoxO protein caused by reduced phosphorylation by the insulin/PI3K/PDK/AKT cascade during fasting (Figure 7I).^60^^,^^61^^,^^62^ The other possibility is that increased FoxO binding to the *Ass1* enhancer is caused by elevated amino acid concentrations during fasting (Figures S2J–S2M), as demonstrated in our previous study using a high-protein diet.^48^

The contribution of FoxO transcription factors to amino acid metabolism remains incompletely defined, despite their well-established roles in numerous other metabolic processes. For instance, liver-specific ablation of *FoxO1*, *FoxO3*, and *FoxO4* (L-*FoxO1*,*3*,*4*) has been shown to impair the fasting-induced expression of glucose-6-phosphatase and prevent the repression of glucokinase, highlighting their importance in glucose homeostasis during nutrient deprivation.^63^ However, elucidating their connection to *Ass1* regulation is complicated by compensatory mechanisms. In the available microarray dataset (GSE60527/GPL6096/6768261), *Ass1* expression levels remained unchanged (13.27 in both hepatocyte-specific *FoxO1*,*3*,*4* knockout and littermate control), suggesting that long-term FoxO depletion may trigger compensatory transcriptional programs that obscure functional relationships. Such compensation is a well-documented phenomenon in gene knockout models, particularly involving transcription factors, and often leads to misinterpretation of regulatory mechanisms. Comparative studies have demonstrated that knockouts and knockdowns can produce markedly different phenotypes across species, including mice.^64^^,^^65^ For example, in mice lacking SREBP-1, hepatic SREBP-2 expression is upregulated to compensate for the genetic loss of SREBP-1.^66^ In this context, knockdown strategies, by inducing acute and partial gene suppression, may provide a more accurate representation of direct regulatory effects, as they are less likely to provoke systemic compensatory adaptations.

Most experiments in this study employed combined *FoxO1/3a* knockdown, reflecting the substantial functional redundancy between these isoforms in regulating fasting-responsive hepatic gene programs, as demonstrated by prior genetic studies showing that concurrent ablation of multiple FoxO isoforms is required for full phenotypic penetrance.^63^ While our data regarding *FoxOs* knockdown do not formally resolve whether one isoform contributes quantitatively more than the other to *Ass1* regulation under fasting conditions, our ChIP data demonstrate that both FoxO1 and FoxO3a independently bind the *Ass1* enhancer in a fasting-dependent manner (Figures 7A and 7E), and both isoforms activate the *Ass1* enhancer reporter in cell-based assays (Figures 5B and 5C), consistent with cooperative rather than exclusively dominant regulation.

It is well established that gluconeogenesis and ureagenesis function cooperatively to support sustained amino acid catabolism in the liver, primarily by generating ATP and facilitating nitrogen disposal.^15^ In liver-specific *FoxO1* transgenic mice, previous studies have shown an upregulation of genes involved in amino acid catabolism and gluconeogenesis, highlighting the role of FoxO1 in coordinating these metabolic pathways in the liver.^4^ This coordination ensures efficient processing of amino acids, maintaining metabolic balance during nutrient deprivation. These findings underscore the need to investigate the role of FoxO transcription factors as potential integrators of these pathways through the regulation of amino acid metabolism. In this context, our data demonstrated that hepatic FoxO depletion leads to partial disruption of the urea cycle and a significant reduction in blood glucose levels (Figure 1). To determine whether ASS1 mediates the regulatory effect of FoxO on urea cycle-related amino acid metabolism, we performed hepatic *Ass1* knockdown. Interestingly, *Ass1* depletion produced a phenotype resembling that of *FoxO* knockdown: both resulted in partially impaired urea cycle function, evidenced by altered ornithine and arginine levels, and lowered blood glucose, even though *Pck1* or *G6pc* gene expression remained unchanged (Figure 3). These findings suggest that *Ass1* is a critical direct target of FoxO transcription factors, mediating their role in integrating ureagenesis and gluconeogenesis. Thus, ASS1 may represent a key regulatory node linking nitrogen disposal and glucose production during starvation.

The physiological significance of the FoxO-*Ass1* axis was directly demonstrated by pyruvate and lactate tolerance tests, in which both *FoxO* and *Ass1* knockdown significantly attenuated glucose production *in vivo* (Figures 4A–4D), establishing that both factors are required for efficient gluconeogenesis during fasting. Importantly, *Ass1* knockdown phenocopied the hypoglycemic effect of *FoxO* knockdown despite unaltered *Pck1* and *G6pc* expression (Figures 3L and 3M), indicating that ASS1 contributes to gluconeogenesis through a substrate-supply mechanism. This was formally demonstrated by the rescue experiment, in which *Ass1* overexpression restored glucose production in FoxO-inhibited primary hepatocytes despite partial suppression of *Pck1* and *G6pc* (Figures 4E–4H), demonstrating that ASS1-dependent generation of argininosuccinate and its subsequent conversion to fumarate, malate, and cytosolic oxaloacetate, can compensate for the reduction in FoxO-driven gluconeogenic gene transcription. This interpretation is consistent with previous studies highlighting the importance of urea cycle-derived fumarate in supporting gluconeogenic carbon flux during starvation.^8^^,^^10^^,^^11^^,^^26^

The coupling between ureagenesis and gluconeogenesis is physiologically essential during nutrient deprivation, when amino acids serve as a primary energy source.^5^^,^^24^ This coordination allows nitrogen to be safely eliminated via the urea cycle, while the resulting carbon skeletons are directed toward glucose production to maintain systemic energy homeostasis.^10^ A key point of intersection between these two pathways occurs through the conversion of aspartate to fumarate, alongside the transformation of citrulline to arginine.^8^ Although oxaloacetate cannot directly traverse the mitochondrial membrane, it can reach the cytosol after transamination to aspartate. This aspartate is then reconverted to oxaloacetate via fumarate and malate, a process that requires ASS1 activity.^47^ Our data support the view that, in the liver, the dominant net route by which aspartate-derived carbon contributes to cytosolic oxaloacetate proceeds through the urea cycle.^8^ Aspartate entry via ASS1, followed by argininosuccinate cleavage to fumarate, enables the return of carbon skeletons to the malate-oxaloacetate pool. In contrast, the reversible aspartate aminotransferase reaction contributes little net oxaloacetate production under steady-state conditions, despite high bidirectional flux.

Clinical evidence from inborn errors of metabolism underscores the physiological coupling between the urea cycle and gluconeogenesis, particularly under fasting conditions. For instance, in pyruvate carboxylase deficiency, impaired conversion of pyruvate to oxaloacetate disrupts both the TCA cycle and gluconeogenesis, leading to fasting-induced hypoglycemia.^67^ Crucially, oxaloacetate is also required for generating aspartate, a key substrate in the urea cycle. Its deficiency reduces argininosuccinate synthesis, resulting in impaired ureagenesis and hyperammonemia, highlighting how a primary gluconeogenic defect can secondarily impair nitrogen disposal.^68^ Conversely, in citrin (AGC2) deficiency, defective mitochondrial export of aspartate and malate reduces cytosolic availability of both substrates, limiting urea cycle activity and simultaneously impairing gluconeogenesis.^69^ This dual impact leads to recurrent hyperammonemia, hypoglycemia, and citrullinemia, caused by insufficient aspartate for urea synthesis and diminished fumarate generation for oxaloacetate production and subsequently gluconeogenesis.^70^ Together, these disorders illustrate the bidirectional metabolic interdependence between ureagenesis and gluconeogenesis, particularly under conditions of nutrient deprivation.

In conclusion, this study identifies the FoxO-*Ass1* axis as the transcriptional mechanism that coordinates ureagenesis to gluconeogenesis during fasting. We demonstrate that FoxO transcription factors directly regulate *Ass1* through a functional enhancer element, independently of KLF15, and that ASS1 is required for efficient glucose production from gluconeogenic substrates. By establishing ASS1 as a functional mediator that coordinates nitrogen disposal with gluconeogenic carbon flux, our findings reveal a previously unrecognized regulatory node that integrates hepatic amino acid metabolism and energy homeostasis during nutrient deprivation.

### Limitations of the study

Although a limitation of the present study is that we did not directly trace ASS1-derived carbon through the fumarate-malate-oxaloacetate pathway, our findings nonetheless identify the FoxO-Ass1 axis as a previously unrecognized transcriptional mechanism coordinating ureagenesis and gluconeogenesis during fasting.

## Resource availability

### Lead contact

Further information and requests for resources and reagents should be directed to and will be fulfilled by the lead contact, Naoya Yahagi (nyahagi-tky@umin.ac.jp).

### Materials availability

Plasmids and adenoviral constructs newly generated in this study are available from the lead contact upon reasonable request, subject to completion of an appropriate materials transfer agreement.

### Data and code availability

• This paper does not report newly generated standardized datasets.
• The previously published FoxO1 ChIP-seq dataset analyzed in this study is publicly available through the Gene Expression Omnibus under accession no. GSM3381273 and was accessed through ChIP-Atlas.
• The previously published hepatocyte-specific FoxO1/3/4 knockout liver microarray dataset analyzed in this study is publicly available through the Gene Expression Omnibus under accession no. GSE60527.
• All other data reported in this paper will be shared by the lead contact upon reasonable request.
• This paper does not report original code.
• Any additional information required to reanalyze the data reported in this paper is available from the lead contact upon reasonable request.

## Acknowledgments

We thank Prof. Mukesh Jain (Case Western Reserve University) for kindly providing us with the *Klf15* knockout mouse. This work was supported by 10.13039/501100001700MEXT/10.13039/501100001691JSPS KAKENHI grant nos. 23116006 (Grant-in-Aid for Scientific Research on Innovative Areas: Crosstalk of transcriptional control and energy pathways by hub metabolites), 23K24760 (Grant-in-Aid for Scientific Research (B)), and 24K22110 (Grant-in-Aid for Challenging Exploratory Research) (to N.Y.). This research was also supported by 10.13039/100009619AMED under grant no. JP23gm1710008 (AMED-CREST) and JP23rea522010 (Healthcare Social Implementation Infrastructure Development Project) (to N.Y.). It was also supported by 10.13039/501100001700MEXT/10.13039/501100001691JSPS KAKENHI grant no. 25K24300 (Research Activity Start-up) (to S.K.).

## Author contributions

S.K. and N.Y. conceived the experiments. S.K. performed the experiments under the guidance of Y.T. and analyzed the data together with N.Y. S.K. and N.Y. co-wrote the paper. All authors discussed the results and commented on the manuscript.

## Declaration of interests

The authors declare no competing financial and non-financial interests.

## STAR★Methods

### Key resources table

**Table:** 

REAGENT or RESOURCE	SOURCE	IDENTIFIER
**Antibodies**		

Mouse monoclonal anti-ASS1 (WB)	Santa Cruz Biotechnology	Cat#sc-365475; RRID: AB_10847087
Mouse monoclonal anti-GAPDH (WB)	Santa Cruz Biotechnology	Cat#sc-32233; RRID: AB_627679
Mouse monoclonal anti-β-Actin (WB)	Santa Cruz Biotechnology	Cat#sc-8432; RRID: AB_626630
Goat polyclonal anti-HNF-4α (WB)	Santa Cruz Biotechnology	Cat#sc-6556; RRID: AB_2117025
Rabbit polyclonal anti-FoxO1 (WB, ChIP, CUT&Tag)	Cell Signaling Technology	Cat#2880; RRID: AB_2106495
Rabbit polyclonal anti-FoxO3a (WB)	Cell Signaling Technology	Cat#2497; RRID: AB_836876
Rabbit monoclonal anti-FoxO3a (ChIP)	Cell Signaling Technology	Cat#12829; RRID: AB_2636990
Mouse monoclonal anti-Lamin A/C (WB)	Santa Cruz Biotechnology	Cat#sc-376248; RRID: AB_10991536
Normal rabbit IgG (ChIP/CUT&Tag)	Sino Biological Inc.	Cat#CR1; RRID: AB_3073921
HRP-conjugated goat anti-mouse IgG (WB)	Cell Signaling Technology	Cat#7076; RRID: AB_330924
HRP-conjugated goat anti-rabbit IgG (WB)	Cell Signaling Technology	Cat#7074; RRID: AB_2099233
HRP-conjugated donkey anti-goat IgG (WB)	Invitrogen	Cat#A15999; RRID: AB_2534673

**Bacterial and virus strains**		

Adenovirus: Ad-*LacZ*i	Takeuchi et al.^38^	N/A
Adenovirus: Ad-*FoxO1,3a*i	Takeuchi et al.^38^	N/A
Adenovirus: Ad-GFP	Takeuchi et al.^38^	N/A
Adenovirus: Ad-FoxODN	Takeuchi et al.^38^	N/A
Adenovirus: Ad-*Ass1*i-1	Karkoutly et al.^48^	N/A
Adenovirus: Ad-*Ass1*i-2	Karkoutly et al.^48^	N/A
Adenovirus: Ad-*Ass1*	This paper	N/A
Adenovirus: Ad-*Ass1*-Enhancer-Luc (WT & Mut)	This paper	N/A
E. coli: Strain DH5α	TOYOBO	Cat#DNA-903F

**Biological samples**		

Mouse liver tissue (ICR and Klf15^+/+^/Klf15^−/−^ C57BL/6J)	This paper	N/A
Mouse plasma (ICR and Klf15^+/+^/Klf15^−/−^ C57BL/6J)	This paper	N/A

**Chemicals, peptides, and recombinant proteins**		

D-Luciferin potassium salt	Wako Chemicals (FUJIFILM)	Cat#126–05116
Lipofectamine 3000 Transfection Reagent	Thermo Fisher Scientific	Cat#L3000-015
Reporter Lysis Buffer	Promega	Cat#E397A
Glutathione Sepharose beads	Amersham Biosciences	Cat#17–0756–01
Firefly luciferase assay reagent (Pikkagene)	Toyo B-Net Bio	Cat#PGL5500
Dynabeads magnetic beads (Protein G)	Thermo Fisher Scientific	Cat#10004D
Sepasol-RNA I Super G	Nacalai Tesque	Cat#09379–55
Collagenase II	Worthington	Cat#LS004176
Recombinant GST protein	This paper	N/A
Recombinant GST-FoxO1-DBD fusion protein	This paper	N/A

**Critical commercial assays**		

ReverTra Ace qPCR RT Master Mix	TOYOBO	Cat#FSQ-201
KAPA SYBR Fast qPCR Kit	NIPPON Genetics	Cat#KK4602
PrimeSTAR GXL DNA Polymerase	TaKaRa Bio	Cat#R050A
In-Fusion HD Cloning Kit	TaKaRa Bio	Cat#071320
PrimeSTAR Mutagenesis Basal Kit	TaKaRa Bio	Cat#R046A
CUT&Tag-IT™ Assay Kit – Tissue	Active Motif	Cat#53170
LabAssay™ Glucose Kit	FUJIFILM Wako	Cat#291–94001
Renilla Luciferase Assay System	Promega	Cat#E2820

**Deposited data**		

FoxO1 ChIP-seq dataset, unfed mouse liver	Kalvisa et al.^51^	GSM3381273
Hepatocyte-specific *FoxO1,3,4* KO liver microarray dataset	Haeusler et al.^63^	GSE60527

**Experimental models: Cell lines**		

Human: HEK293 embryonic kidney cells	ATCC	RRID:CVCL_0045
Human: HepG2 hepatoma cells	ATCC	RRID:CVCL_0027

**Experimental models: Organisms/strains**		

Mouse: ICR	Japan SLC, Inc.	N/A
Mouse: Klf15KO C57BL/6J background	Fisch et al.^71^	N/A

**Oligonucleotides**		

Primers for Q-RT PCR	see Table S1	N/A
Primers for PCR	see Table S2	N/A
Probes for EMSA	see Table S3	N/A
Primers for ChIP Q-PCR	see Table S4	N/A
Primers for CUT&Tag qPCR	see Table S5	N/A

**Recombinant DNA**		

Plasmid: *Ass1*-Enhancer-Luc (full-length, fragments Fr1, Fr2, Fr3, Fr1A, Fr1B, and mutated versions)	This paper	N/A
Plasmid: pRL-SV40	Promega	Cat#E2231
Plasmid: FoxO1 expression vector	Yahagi et al.^55^	N/A
Plasmid: FoxO3a expression vector	Yahagi et al.^55^	N/A
Plasmid: pGEX-4T1-GST-FoxO1-DBD	Takeuchi et al.^36^	N/A
Plasmid: pGEX-4T	Amersham Biosciences	Cat#28–9545–49
Plasmid: pENTR4	Thermo Fisher Scientific	Cat#11818–010
Plasmid: pENTR4-Luc	Takeuchi et al.^38^	N/A
Plasmid: pAd/CMV/V5-DEST	Thermo Fisher Scientific	Cat#V49320
Plasmid: pENTR/U6	Thermo Fisher Scientific	Cat#K494500

**Software and algorithms**		

LIVING IMAGE™ software	PerkinElmer	RRID:SCR_014247
ImageJ 1.50i	NIH	RRID:SCR_003070
QuantStudioTM Design and Analysis Software v1.5.1	Thermo Fisher Scientific	N/A
UCSC Genome Browser on Mouse NCBI37/mm9 Assembly	UCSC	RRID:SCR_005780
Python (v3.12.3)	Python Software Foundation	RRID:SCR_008394
pandas (v3.0.2)	pandas development team	RRID:SCR_018214
NumPy (v2.4.4)	NumPy developers	RRID:SCR_008633
SciPy (v1.17.1)	SciPy developers	RRID:SCR_008058
statsmodels (v0.14.6)	statsmodels developers	N/A

**Other**		

IVIS™ Imaging System	PerkinElmer	RRID:SCR_018621
QuantStudio™ 5 Real-Time PCR System	Thermo Fisher Scientific	N/A
Bioruptor® 2 sonicator	Sonicbio (Diagenode)	N/A
Wallac ARVO SX 1420 luminometer	PerkinElmer	N/A
Hitachi High-Tech L-8080 amino acid analyzer	Hitachi High-Tech Corporation	N/A

### Experimental model and study participant details

#### Animals

Male ICR mice aged between 5 and 7 weeks were purchased from Japan SLC, Inc. (Shizuoka, Japan). The *Klf15*^−/−^ (*Klf15*KO) backcrossed into C57BL/6J strain mice was kindly gifted by Prof. Jain MK.^71^ Controls were C57BL/6J *Klf15*^+/+^ (wild-type) mice. The experiments using ICR were performed between 6 and 8 weeks of age, and the experiments using *Klf15*^+/+^ and *Klf15*^−/−^mice were performed at 5–6 months of age. The animals were housed in a temperature-controlled environment with a 12-h light/12-h dark cycle and provided free access to standard laboratory diet and water. After introducing the standard laboratory diet (Cat#MF; Oriental Yeast, Tokyo, Japan; consisted of 25.5% of energy from protein, 61.5% from carbohydrates, and 13% from fat), mice were fasted for 24 h, and blood glucose levels were measured from tail vein blood using a handheld glucometer before sacrifice. Mice were sacrificed during the early light phase in either fasted or *ad libitum*-fed states (free access to food). For pyruvate and lactate tolerance testing, 5-7-week male mice fed with normal chow were fasted 24h, followed by intraperitoneal injection of sodium pyruvate (2 g kg^−1^ body weight) or sodium lactate (2 g kg^−1^ body weight), blood glucose was measured by tail bleeding at 0, 30, 60, 90, and 120 min after injection. Regarding experimental design, mice of the same age and comparable body weight were randomly assigned to experimental groups prior to adenoviral injection, housed under identical conditions, and sacrificed at the same time in the light cycle to minimize circadian variability. Complete observer blinding was not implemented. All animal procedures were performed following the protocol approved by the Jichi Medical University Animal Care and Use Committee, approval number 23068–04, and were conducted in accordance with relevant institutional and national regulatory guidelines and standards. The experiments were repeated at least twice to correct the bias for each experimental environment.

#### Cell culture

HEK293 human embryonic kidney cells (RRID:CVCL_0045) and HepG2 human hepatoma cells (RRID: CVCL_0027) were distributed from ATCC (American Type Culture Collection, Manassas, VA, USA), and cultured in DMEM containing 25 mM glucose, 100 U/mL penicillin, and 100 μg/mL streptomycin sulfate supplemented with 10% CCS or 10% FBS. All cell lines have been authenticated by our facility’s authentication protocol within the last 3 years, and all experiments were performed with mycoplasma-free cells.

#### Primary cell cultures

Primary hepatocytes were isolated from male ICR mice aged between 5 and 7 weeks. Culture media and growth conditions varied according to the experimental stage and are described in detail in the relevant STAR Methods section.

### Method details

#### Preparation and transduction of recombinant adenoviruses

Adenoviral vectors encoding *LacZ*i, *FoxO*i, GFP (Ad-GFP), as well as two mouse *Ass1*-specific shRNA constructs (*Ass1i*-1 and *Ass1i*-2), have been described previously.^38^^,^^48^ A dominant-negative form of FoxO1/3/4 (FoxODN) was generated from a *FoxO1* cDNA fragment lacking the C-terminal transactivation domain, which was amplified by PCR using the TFEL-*FoxO1* plasmid as a template.^38^ Mouse *Ass1* cDNA was subcloned into the pENTR4 entry vector, and an adenoviral *Ass1* overexpression construct was generated through homologous recombination with the pAd/CMV/V5-DEST vector (Invitrogen). The DNA fragment of mouse *Ass1* enhancer region containing the FoxO binding site was amplified by PCR using PrimeSTAR GXL DNA Polymerase (Cat#R050A, TaKaRa Bio, Tokyo, Japan), using mouse genomic DNA as template and inserted into multiple cloning site on the pENTR4-Luc Gateway entry vector linked to firefly luciferase reporter with *Ass1* native promoter (*Ass1*-Enhancer-Luc) by infusion technique using In-Fusion HD Cloning Kit (Cat#071320, TaKaRa Bio, Tokyo, Japan) according to the manufacturer’s protocol. Mutated *Ass1*-Enhancer-Luc plasmid was generated using PrimeSTAR Mutagenesis Basal Kit (Cat# R046A, TaKaRa Bio, Tokyo, Japan). Primer sets are listed in Table S2. Adenoviral constructs were generated by homologous recombination between the entry vector and the pAd promoterless vector (Thermo Fisher Scientific, Waltham, MA, USA). Recombinant adenoviruses were propagated in HEK293 cells. Recombinant adenoviruses were then purified by CsCl gradient centrifugation, and titers were quantified as described previously.^36^^,^^38^^,^^52^^,^^72^ For animal experiments, adenoviruses were injected intravenously into ICR male mice from subclavian vein at the following doses: for *LacZ*i/*FoxO*i, 20 × 10^8 P.F.U.; for GFP/FoxODN, 7.5 × 10^8 P.F.U.; *Ass1*-enhancer-Luc, 5 × 10^8 P.F.U.; *LacZ*i/*Ass1*i 20 × 10^8 P.F U. For primary hepatocytes the following doses were used: for Ad-FoxODN, 10 m.o.i.; for Ad-*Ass1*, 1 m.o.i. (1 P.F U. is equal to 1000 optical particles of adenovirus).

#### Amino acid measurement

Amino acid measurements were performed as previously described.^48^ In brief, for plasma amino acids measurement, blood samples were collected from vena cava under a three-type mixed anesthesia consisting of medetomidine, midazolam, and butorphanol. Protein components were precipitated in the collected plasma samples by mixing them with equal amounts of 3% ice-cold sulfosalicylic acid. After centrifugation the supernatant was collected. The sample pH was adjusted to pH 2–3 and then filtered using a centrifuge filter (Cat#UFC30HV00, Millipore, Merck KGaA, Darmstadt, Germany). The whole process was performed on ice. The amino acid composition was measured using Hitachi-Hightech (LA8080).

For liver amino acids measurement, mouse liver samples were homogenized in 3% ice-cold sulfosalicylic acid to precipitate proteins. Later steps are as explained in plasma amino acids measurement.

#### RNA extraction and quantitative reverse transcription PCR (Q-RT PCR)

Total RNA was extracted from 50 to 100 mg of mouse liver using Sepasol-RNA I Super G (Cat# 09379–55; Nacalai Tesque, Kyoto, Japan) according to the manufacturer’s instructions. The extracted RNA (500 ng) was reverse transcribed in a volume of 5 μL and converted to cDNA using the ReverTra Ace qPCR RT Master Mix (Cat# FSQ-201, TOYOBO, Osaka, Japan). Real-time PCR was performed using KAPA SYBR Fast qPCR Kit (Cat# KK4602, NIPPON Genetics, Tokyo, Japan) on a QuantStudio^TM^ 5 Real-Time PCR System (Thermo Fisher Scientific, Waltham, MA, USA) and quantified by the standard curve method with cDNA as the template. After amplification by PCR, samples containing the product with the correct Tm value were taken based on the melting curve plot for each sample. Primer sets are listed in Table S1. Cyclophilin A was used as an internal reference to correct gene expression level for each sample.

#### Isolation and culture of primary hepatocytes

Primary hepatocytes were isolated from male ICR mice aged between 5 and 7 weeks with collagenase perfusion method. Mice were anesthetized, and the portal vein was cannulated with a 24-gauge cannula. HBSS containing 0.5 mM EDTA was perfused to chelate calcium, and then HBSS containing 5 mM CaCl2 and 1 mg/mL Collagenase Type II (Cat#LS004176, Worthington Biochemical Corporation, Lakewood, NJ, USA) was perfused to dissociate extracellular matrix of the liver. After the liver dissection, cells were filtered with 40 mm mesh cell strainer, and hepatocytes were purified by gradient centrifugation method. Hepatocytes were suspended in DMEM containing 25 mM glucose, 100 nM insulin, 10 nM dexamethasone, 100 U/mL penicillin, and 100 mg/mL streptomycin sulfate supplemented with 10% FBS and plated in 6-well plates at 1 × 10^6^ cells/cm^2^ for 3h. For adenovirus transduction, the medium was changed to DMEM containing the indicated adenoviruses, and 25 mM glucose, 100 U/mL penicillin, and 100 mg/mL streptomycin sulfate supplemented with 10% FBS for 8h. For the glucose production assay medium was replaced with starvation medium DMEM containing 5 mM glucose, 100 U/mL penicillin, and 100 mg/mL streptomycin sulfate without FBS overnight, then finally replaced with DMEM, no glucose, no phenol red containing 20 mM sodium lactate, 2 mM sodium pyruvate. Medium was collected and glucose was measured with Mutarotase–GOD method (LabAssay^TM^ Glucose, Cat#291–94001, FUJIFILM Wako, Japan).

#### Plasmid construction

For constructing *Ass1*-enhancer firefly luciferase reporter plasmid, a 2.1 kbp-region of the 5′-flanking sequence at 7 kbp upstream from transcriptional start site on the mouse *Ass1* gene were chosen as the enhancer sequence for our experiments. Figure S5 shows UCSC Genome Browser tracks for H3K4me1, H3K27ac, and H3K9ac. The estimated enhancer site and its smaller fragments were constructed by amplifying genomic DNA by PCR using PrimeSTAR GXL DNA Polymerase (Cat#R050A, TaKaRa Bio, Tokyo, Japan) and linked to luciferase reporter gene by infusion technique using In-Fusion HD Cloning Kit (Cat#071320, TaKaRa Bio, Tokyo, Japan) according to the manufacturer’s protocol. Mutated *Ass1*-enhancer firefly luciferase reporter plasmids were generated using PrimeSTAR Mutagenesis Basal Kit (Cat# R046A, TaKaRa Bio, Tokyo, Japan). Used primers are listed in Table S2.

#### Luciferase assay

For luciferase assay as described previously^38^ cells were seeded in 48-well plate to 20% confluency and transfected using Lipofectamine 3000 reagent (Cat# L3000-015, Thermo Fisher Scientific, Waltham, MA, USA) according to the manufacturer’s protocol. Cells were transfected with *FoxO1* and *FoxO3a* expression plasmids,^55^ *Ass1*-enhancer firefly luciferase reporter plasmids and Renilla luciferase reporter plasmid (pRL-SV40; Promega, Madison, WI, USA) after adjusting total amounts of transfected DNA with empty plasmid. After 48 h cells were lysed with 100 μL of Reporter Lysis Buffer (Cat# E397A, Promega, Madison, WI, USA) and centrifuged. The supernatant was mixed with a luminometer with a firefly luciferase assay reagent (Cat# PGL5500, Pikkagene, Toyo Bnet bio, Tokyo, Japan) and the firefly luciferase activity was measured using a Wallac ARVO SX 1420 luminometer (PerkinElmer, Shelton, CT, USA). Renilla luciferase activity was measured with Renilla Luciferase Assay System (Cat# E2820, Promega, Madison, WI, USA) according to the manufacturer protocol. The Renilla luciferase activities were used to normalize transfection efficiencies.

#### Electrophoretic mobility shift assay (EMSA)

EMSA were performed as described previously.^72^ In brief, the DNA probes were prepared by annealing two oligonucleotides, labeled with [α-^32^P] dCTP by filling in the 5′-overhangs with Klenow DNA polymerase, and purified on Sephadex G-50 columns. EMSA probes are listed in Table S3. A DNA fragment encoding the amino acid sequence (157 aa to 268 aa) containing the mouse FoxO1 DNA binding domain was inserted into the MCS of pGEX-4T1 to prepare a GST-fusion protein (GST-FoxO1-DBD). The labeled DNA probes were incubated with GST and GST-FoxO1-DBD in binding buffer (10 mM HEPES at pH 7.8, 50 mM KCl, 1 mM EDTA, 5 mM MgCl_2_, 10% glycerol, 5 mM dithiothreitol, and 0.4 μg/mL poly(dI-dC)), for 30 min on ice. The DNA-protein complex was analyzed on 4.6% polyacrylamide gels in TBE buffer.

GST and GST fusion proteins were expressed in *E. coli* (DH5α) using pGEX-4T (Amersham BioSciences, Buckinghamshire, UK) and purified using glutathione Sepharose beads (Cat# 17–0756–01, Amersham BioSciences, Buckinghamshire, UK) by standard method as described previously.^36^

#### *In vivo* imaging of luciferase activity

*In vivo* imaging was performed as described previously.^36^^,^^52^^,^^53^^,^^54^ After 4 days of the adenovirus transduction, animals were fasted for 24-h from the early dark phase. At each condition, D-Luciferin potassium salt (Cat# 126–05116, Wako Chemicals, Tokyo, Japan) dissolved in PBS at a concentration of 7.5 mg/mL was intraperitoneally injected at a dose of 10 mL/kg into mice and the luminescence in the liver was captured using an IVIS^TM^ Imaging System (PerkinElmer, Waltham, MA, USA). Relative photon emissions over the liver region was quantified using LIVING IMAGE^TM^ software (PerkinElmer). Hepatic transduction efficiency was determined based on the quantification of adenoviral DNA in the liver using a previously described Q-PCR method,^73^ and the result of quantification was used to normalize the *in vivo* imaging of luciferase activity. Two paired data from the same animal on different nutritional conditions (i.e., before and after fasting) were obtained and the ratio between the two quantities was used to cancel the variations in hepatic transduction efficiencies.

#### Chromatin immunoprecipitation (ChIP) assay

Chromatin immunoprecipitation (ChIP) assays using mouse liver were performed as described previously.^38^^,^^48^ Briefly, 100 mg of liver tissue samples from the fasted and Ad libitum-fed mice were minced in 1 mL of PBS and cross-linked in 1.5% formaldehyde for 15 min at room temperature. Fixed samples were homogenized and then subjected to sonication with Bioruptor2 (Sonicbio, Kanagawa, Japan) for DNA fragmentation. After centrifugation, supernatant was diluted to 6 mL with dilution buffer (50 mM Tris-HCl at pH 8.0, 167 mM NaCl, 1 mM EDTA, 1.1% Triton X-100, and 0.1% sodium deoxycholate) and then 1 mL of total volume was used for immunoprecipitation with 1 μg of anti-FoxO1 (Cat#2880, Cell Signaling Technology, Danvers, MA, USA), anti-FoxO3a (Cat#12829, Cell Signaling Technology, Danvers, MA, USA), or control IgG (Cat# CR1, Sino Biological Inc.) bound to 30 μL of Dynabeads magnetic beads (Cat# 10004D, Thermo Fisher Scientific, Waltham, MA, USA) and rotated overnight at 4°C. Fifty microliter of total volume was used for input sample. The complexes were washed with low-salt wash buffer (50 mM Tris-HCl at pH 8.0, 150 mM NaCl, 1 mM EDTA, 1% Triton X-100, 0.1% SDS, and 0.1% sodium deoxycholate), high-salt wash buffer (50 mM Tris-HCl at pH 8.0, 500 mM NaCl, 1 mM EDTA, 1% Triton X-100, 0.1% SDS, and 0.1% sodium deoxycholate), LiCl wash buffer (10 mM Tris-HCl at pH 8.0, 0.25 M LiCl, 1 mM EDTA, 0.5% NP40, and 0.5% sodium deoxycholate), and TE buffer. DNA-protein complex was eluted by incubation with elution buffer (1% SDS, 0.1 M NaHCO_3_) for 15 min at room temperature and then incubated with 200 mM NaCl overnight at 65°C for reverse crosslinking. DNA-protein complex was treated with 200 μg/mL proteinase K, and chromatin DNA was purified with phenol-chloroform, eluted in TE buffer, and subjected to Q-PCR analysis. From reverse crosslinking step, the input sample was also subjected to the same procedure. Q-PCR was performed using the same method as Q-RT PCR and quantified by standard curve method with input DNA samples. The primer sets are listed in Table S4.

#### Total liver lysate

For total liver lysate, 20 mg of liver tissues collected from 3 to 4 mice were pooled and homogenized in 1 mL lysis buffer (50 mM Tris-HCl at pH 7.5, 137 mM NaCl, 1 mM EDTA, 1% Triton-X, protease inhibitors). After centrifugation at 15,000 rpm for 10 min at 4°C, the supernatant was used as a total liver lysate.

#### Liver nuclear extraction

Liver nuclear extraction was performed as previously described.^74^ In brief, 1.5 g of liver tissues collected from 3 to 4 mice were pooled and homogenized in 15 mL of buffer A (10 mM HEPES at pH 7.6, 25 mM KCl, 1 mM EDTA, 2 M sucrose, 10% glycerol, 0.15 mM spermine, 2 mM spermidine, protease inhibitors). The sample was filtered with sterile gauze (Kawamoto Corporation) and layered on 15 mL of buffer A in a polypropylene centrifuge tube (Beckman coulter, Brea, CA, USA). The tube was centrifuged at 24,000 rpm for 90 min at 4°C. The pellet was suspended in 800 μL of buffer B (10 mM HEPES at pH 7.6, 100 mM KCl, 2 mM MgCl_2_, 1 mM EDTA, 1 mM DTT, 10% glycerol, protease inhibitors) and centrifuged at 89,000 rpm for 20 min at 4°C. The supernatant was used as a nuclear extract.

#### Western blotting

Western blotting was performed as previously described.^75^^,^^76^ In brief, after SDS-PAGE, proteins were transferred onto nitrocellulose membrane (Hybond ECL, Amersham BioSciences, Buckinghamshire, UK). Following protein transfer, membranes were stained with Ponceau S solution (0.1% w/v in 5% acetic acid) to verify transfer efficiency and confirm equal protein loading prior to immunodetection. ASS1, GAPDH, β-Actin, HNF4α, FoxO1, FoxO3a, and Lamin were detected using a 1:1000 dilution mouse anti-ASS1 (Cat#sc-365475, Santa Cruz Biotechnology, Dallas, TX, USA), mouse anti-GAPDH (Cat#sc-32233, Santa Cruz Biotechnology, Dallas, TX, USA), mouse anti-Actin (Cat# sc-8432, Santa Cruz Biotechnology, Dallas, TX, USA), goat anti-HNF-4α (Cat# sc-6556, Santa Cruz Biotechnology, Dallas, TX, USA), rabbit anti-FoxO1 (Cat#2880, Cell Signaling Technology, Danvers, MA, USA), rabbit anti-FoxO3a (Cat#2497, Cell Signaling Technology, Danvers, MA, USA), and mouse anti-LaminA/C (Cat#sc-376248, Santa Cruz Biotechnology, Dallas, TX, USA), in TBS buffer (20 mM Tris-HCl at pH 7.6 and 140 mM NaCl) containing 0.2% Tween 20 and 5% skimmed milk. Bound antibodies were detected with a horseradish peroxidase-coupled anti-mouse IgG secondary antibody (Cat#7076, Cell Signaling Technology, Danvers, MA, USA), anti-rabbit IgG secondary antibody (Cat#7074, Cell Signaling Technology, Danvers, MA, USA) and anti-goat IgG secondary antibody (Cat#A15999, Invitrogen, Waltham, MA, USA) and visualized using anti-ECL chemiluminescent substrates (Cat# NEL104001EA, Revvity Health Sciences Inc.).

#### CUT&Tag assay

CUT&Tag assays were performed on snap-frozen liver samples from ICR mice (6–8 weeks of age) using the CUT&Tag-IT Assay Kit – Tissue (Active Motif, Cat# 53170), according to the manufacturer’s instructions. Chromatin was tagmented using an anti-FoxO1 antibody (Cat#2880, Cell Signaling Technology, Danvers, MA, USA) or normal rabbit IgG (Cat# CR1, Sino Biological Inc.) as a negative control. Because CUT&Tag typically produces short fragments of less than 200 bp, locus-specific enrichment was quantified by qPCR using primers in closer proximity to the confirmed FOXO-binding site within the *Ass1* enhancer, the *Pck1* promoter FoxO-binding site, and within-locus negative control primer flanking the *Ass1* gene body, with a common i7 reverse primer. The use of the i7 reverse primer rather than a locus-specific reverse primer as in conventional ChIP, further ensures that only antibody-captured, tagmented fragments are detected, thereby overcoming the additional CUT&Tag limitation of genomic DNA leakage from permeabilized nuclei and excluding any signal derived from non-specifically released background DNA. The primer sets are listed in Table S5.

### Quantification and statistical analysis

Data are expressed as means ± S.E.M unless otherwise indicated. Statistical analyses were performed in Python (v3.12.3) using the pandas (v3.0.2), NumPy (v2.4.4), SciPy (v1.17.1), and statsmodels (v0.14.6) packages. Comparisons between two independent groups were performed using an unpaired two-tailed Student’s *t* test. Paired two-tailed Student’s t-tests were used for paired IVIS measurements obtained from the same animals before and after fasting. Experiments involving three or more groups were analyzed using one-way ANOVA followed by Dunnett’s multiple-comparisons test when multiple experimental groups were compared with a common control, or Tukey’s honestly significant difference test when all pairwise comparisons were performed. Factorial experiments involving two independent variables were analyzed using two-way ANOVA, including the main effects of each factor and their interaction, followed by Šídák’s multiple-comparisons test for prespecified pairwise comparisons. Welch’s *t* test (unequal variance) with Šídák correction for multiple comparisons was used for datasets with substantially different group variances. Technical replicates were averaged before statistical analysis, and inferential statistical analyses were performed using independent biological replicates. Differences were considered significant if *p* < 0.05 (∗*p* < 0.05, ∗∗*p* < 0.01, ∗∗∗*p* < 0.001). All statistical details, including the specific test used, exact n and its definition, and the center/dispersion measures (mean ± SEM), are reported in the corresponding figure legends.

## References

[1] Rose A.J. (2019). Amino Acid Nutrition and Metabolism in Health and Disease. Nutrients.

[2] Chou C.J., Affolter M., Kussmann M. (2012). A nutrigenomics view of protein intake: Macronutrient, bioactive peptides, and protein turnover. Prog. Mol. Biol. Transl. Sci..

[3] Ling Z.-N., Jiang Y.-F., Ru J.-N., Lu J.-H., Ding B., Wu J. (2023). Amino acid metabolism in health and disease. Signal Transduct. Target. Ther..

[4] Torres N., Tobón-Cornejo S., Velazquez-Villegas L.A., Noriega L.G., Alemán-Escondrillas G., Tovar A.R. (2023). Amino Acid Catabolism: An Overlooked Area of Metabolism. Nutrients.

[5] Paulusma C.C., Lamers W.H., Broer S., van de Graaf S.F.J. (2022). Amino acid metabolism, transport and signaling in the liver revisited. Biochem. Pharmacol..

[6] Guevara-Cruz M., Vargas-Morales J.M., Méndez-García A.L., López-Barradas A.M., Granados-Portillo O., Ordaz-Nava G., Rocha-Viggiano A.K., Gutierrez-Leyte C.A., Medina-Cerda E., Rosado J.L. (2018). Amino acid profiles of young adults differ by sex, body mass index and insulin resistance. Nutr. Metab. Cardiovasc. Dis..

[7] Berg J.M., Stryer L., Tymoczko J.L., Gatto G.J. (2015).

[8] Jungas R.L., Halperin M.L., Brosnan J.T. (1992). Quantitative analysis of amino acid oxidation and related gluconeogenesis in humans. Physiol. Rev..

[9] Jean C., Rome S., Mathé V., Huneau J.-F., Aattouri N., Fromentin G., Achagiotis C.L., Tomé D. (2001). Metabolic evidence for adaptation to a high protein diet in rats. J. Nutr..

[10] Holeček M. (2023). Roles of malate and aspartate in gluconeogenesis in various physiological and pathological states. Metabolism.

[11] Martín-Requero A., Ciprés G., González-Manchón C., Ayuso M.S., Parrilla R. (1993). Interrelationships between ureogenesis and gluconeogensis in perfused rat liver. Biochim. Biophys. Acta.

[12] Lightbodv H., Kleinman A. (1939). Variations produced by food differences in the concentration of arginase in the livers of white rats. J. Biol. Chem..

[13] Caldovic L., Uchiumi F. (2018). Gene Expression and Control.

[14] Morris J.,S.M. (2002). Regulation of enzymes of the urea cycle and arginine metabolism. Annu. Rev. Nutr..

[15] Watford M. (2003). The urea cycle: Teaching intermediary metabolism in a physiological setting. Biochem. Mol. Biol. Educ..

[16] Schimke R.T. (1962). Adaptive characteristics of urea cycle enzymes in the rat. J. Biol. Chem..

[17] Schimke R.T. (1962). Differential effects of fasting and protein-free diets on levels of urea cycle enzymes in rat liver. J. Biol. Chem..

[18] Hamberg O., Vilstrup H. (1994). Effects of insulin and glucose on urea synthesis in normal man, independent of pancreatic hormone secretion. J. Hepatol..

[19] Grøfte T., Wolthers T., Jensen S.A., Møller N., Jørgensen J.O., Tygstrup N., Ørskov H., Vilstrup H. (1997). Effects of growth hormone and insulin-like growth factor-I singly and in combination on in vivo capacity of urea synthesis, gene expression of urea cycle enzymes, and organ nitrogen contents in rats. Hepatology.

[20] Deguchi K., Ushiroda C., Kamei Y., Kondo K., Tsuchida H., Seino Y., Yabe D., Suzuki A., Nagao S., Iizuka K. (2025). Glucose and Insulin Differently Regulate Gluconeogenic and Ureagenic Gene Expression. J. Nutr. Sci. Vitaminol..

[21] Takiguchi M., Mori M. (1995). Transcriptional regulation of genes for ornithine cycle enzymes. Biochem. J..

[22] Schutz Y. (2011). Protein turnover, ureagenesis and gluconeogenesis. Int. J. Vitam. Nutr. Res..

[23] Gannon M.C., Nuttall F.Q. (2010). Amino acid ingestion and glucose metabolism—a review. IUBMB Life.

[24] Korenfeld N., Finkel M., Buchshtab N., Bar-Shimon M., Charni-Natan M., Goldstein I. (2021). Fasting hormones synergistically induce amino acid catabolism genes to promote gluconeogenesis. Cell. Mol. Gastroenterol. Hepatol..

[25] Voet D., Voet J.G. (2010).

[26] Ipata P.L., Pesi R. (2017). Understanding the interrelationship between the synthesis of urea and gluconeogenesis by formulating an overall balanced equation. Adv. Physiol. Educ..

[27] Lusk G. (1914).

[28] Krebs H.A., Henseleit K. (1932). Untersuchungen uber die Harnstoffbildung im Tierkörper. Klin. Wochenschr..

[29] Krebs H.A., Johnson W.A. (1980). The role of citric acid in intermediate metabolism in animal tissues. FEBS Lett..

[30] Gray S., Wang B., Orihuela Y., Hong E.G., Fisch S., Haldar S., Cline G.W., Kim J.K., Peroni O.D., Kahn B.B., Jain M.K. (2007). Regulation of gluconeogenesis by Krüppel-like factor 15. Cell Metab..

[31] Bieker J.J. (2001). Krüppel-like factors: three fingers in many pies. J. Biol. Chem..

[32] Kaczynski J., Cook T., Urrutia R. (2003). Sp1-and Krüppel-like transcription factors. Genome Biol..

[33] Fan L., Hsieh P.N., Sweet D.R., Jain M.K. (2018). Krüppel-like factor 15: Regulator of BCAA metabolism and circadian protein rhythmicity. Pharmacol. Res..

[34] Jeyaraj D., Scheer F.A.J.L., Ripperger J.A., Haldar S.M., Lu Y., Prosdocimo D.A., Eapen S.J., Eapen B.L., Cui Y., Mahabeleshwar G.H. (2012). Klf15 orchestrates circadian nitrogen homeostasis. Cell Metab..

[35] Mehrazad Saber Z., Takeuchi Y., Sawada Y., Aita Y., Ho M.H., Karkoutly S., Tao D., Katabami K., Ye C., Murayama Y. (2021). High protein diet-induced metabolic changes are transcriptionally regulated via KLF15-dependent and independent pathways. Biochem. Biophys. Res. Commun..

[36] Takeuchi Y., Yahagi N., Aita Y., Murayama Y., Sawada Y., Piao X., Toya N., Oya Y., Shikama A., Takarada A. (2016). KLF15 enables rapid switching between lipogenesis and gluconeogenesis during fasting. Cell Rep..

[37] Teshigawara K., Ogawa W., Mori T., Matsuki Y., Watanabe E., Hiramatsu R., Inoue H., Miyake K., Sakaue H., Kasuga M. (2005). Role of Krüppel-like factor 15 in PEPCK gene expression in the liver. Biochem. Biophys. Res. Commun..

[38] Takeuchi Y., Yahagi N., Aita Y., Mehrazad-Saber Z., Ho M.H., Huyan Y., Murayama Y., Shikama A., Masuda Y., Izumida Y. (2021). FoxO-KLF15 pathway switches the flow of macronutrients under the control of insulin. iScience.

[39] Martins R., Lithgow G.J., Link W. (2016). Long live FOXO: unraveling the role of FOXO proteins in aging and longevity. Aging Cell.

[40] Calissi G., Lam E.W.-F., Link W. (2021). Therapeutic strategies targeting FOXO transcription factors. Nat. Rev. Drug Discov..

[41] Eijkelenboom A., Burgering B.M.T. (2013). FOXOs: signalling integrators for homeostasis maintenance. Nat. Rev. Mol. Cell Biol..

[42] Hall R.K., Yamasaki T., Kucera T., Waltner-Law M., O'Brien R., Granner D.K. (2000). Regulation of phosphoenolpyruvate carboxykinase and insulin-like growth factor-binding protein-1 gene expression by insulin. The role of winged helix/forkhead proteins. J. Biol. Chem..

[43] Schmoll D., Walker K.S., Alessi D.R., Grempler R., Burchell A., Guo S., Walther R., Unterman T.G. (2000). Regulation of glucose-6-phosphatase gene expression by protein kinase Balpha and the forkhead transcription factor FKHR. Evidence for insulin response unit-dependent and -independent effects of insulin on promoter activity. J. Biol. Chem..

[44] Puigserver P., Rhee J., Donovan J., Walkey C.J., Yoon J.C., Oriente F., Kitamura Y., Altomonte J., Dong H., Accili D., Spiegelman B.M. (2003). Insulin-regulated hepatic gluconeogenesis through FOXO1-PGC-1alpha interaction. Nature.

[45] Matsumoto M., Pocai A., Rossetti L., Depinho R.A., Accili D. (2007). Impaired regulation of hepatic glucose production in mice lacking the forkhead transcription factor Foxo1 in liver. Cell Metab..

[46] Matsumoto M., Han S., Kitamura T., Accili D. (2006). Dual role of transcription factor FoxO1 in controlling hepatic insulin sensitivity and lipid metabolism. J. Clin. Investig..

[47] Zhang W., Patil S., Chauhan B., Guo S., Powell D.R., Le J., Klotsas A., Matika R., Xiao X., Franks R. (2006). FoxO1 regulates multiple metabolic pathways in the liver: effects on gluconeogenic, glycolytic, and lipogenic gene expression. J. Biol. Chem..

[48] Karkoutly S., Takeuchi Y., Mehrazad Saber Z., Ye C., Tao D., Aita Y., Murayama Y., Shikama A., Masuda Y., Izumida Y. (2024). FoxO transcription factors regulate urea cycle through Ass1. Biochem. Biophys. Res. Commun..

[49] Xiong X., Tao R., DePinho R.A., Dong X.C. (2013). Deletion of hepatic FoxO1/3/4 genes in mice significantly impacts on glucose metabolism through downregulation of gluconeogenesis and upregulation of glycolysis. PLoS One.

[50] Chaves M., Oyarzún D.A. (2019). Dynamics of complex feedback architectures in metabolic pathways. Automatica.

[51] Kalvisa A., Siersbæk M.S., Præstholm S.M., Christensen L.J.L., Nielsen R., Stohr O., Vettorazzi S., Tuckermann J., White M., Mandrup S., Grøntved L. (2018). Insulin signaling and reduced glucocorticoid receptor activity attenuate postprandial gene expression in liver. PLoS Biol..

[52] Takeuchi Y., Yahagi N., Izumida Y., Nishi M., Kubota M., Teraoka Y., Yamamoto T., Matsuzaka T., Nakagawa Y., Sekiya M. (2010). Polyunsaturated fatty acids selectively suppress sterol regulatory element-binding protein-1 through proteolytic processing and autoloop regulatory circuit. J. Biol. Chem..

[53] Takeuchi Y., Murayama Y., Aita Y., Mehrazad Saber Z., Karkoutly S., Tao D., Katabami K., Ye C., Shikama A., Masuda Y. (2024). GR-KLF15 pathway controls hepatic lipogenesis during fasting. FEBS J..

[54] Murayama Y., Yahagi N., Takeuchi Y., Aita Y., Mehrazad Saber Z., Wada N., Li E., Piao X., Sawada Y., Shikama A. (2019). Glucocorticoid receptor suppresses gene expression of Rev-erbα (Nr1d1) through interaction with the CLOCK complex. FEBS Lett..

[55] Yahagi N., Takeuchi Y. (2021). Genome-wide screening of upstream transcription factors using an expression library. F1000Res..

[56] Kitano H. (2004). Biological robustness. Nat. Rev. Genet..

[57] Haines R.J., Pendleton L.C., Eichler D.C. (2010). Argininosuccinate synthase: at the center of arginine metabolism. Int. J. Biochem. Mol. Biol..

[58] Husson A., Brasse-Lagnel C., Fairand A., Renouf S., Lavoinne A. (2003). Argininosuccinate synthetase from the urea cycle to the citrulline–NO cycle. Eur. J. Biochem..

[59] Anderson G.M., Freytag S.O. (1991). Synergistic activation of a human promoter in vivo by transcription factor Sp1. Mol. Cell Biol..

[60] Brunet A., Bonni A., Zigmond M.J., Lin M.Z., Juo P., Hu L.S., Anderson M.J., Arden K.C., Blenis J., Greenberg M.E. (1999). Akt promotes cell survival by phosphorylating and inhibiting a Forkhead transcription factor. Cell.

[61] Teaney N.A., Cyr N.E. (2023). FoxO1 as a tissue-specific therapeutic target for type 2 diabetes. Front. Endocrinol..

[62] Bideyan L., Nagari R., Tontonoz P. (2021). Hepatic transcriptional responses to fasting and feeding. Genes Dev..

[63] Haeusler R.A., Hartil K., Vaitheesvaran B., Arrieta-Cruz I., Knight C.M., Cook J.R., Kammoun H.L., Febbraio M.A., Gutierrez-Juarez R., Kurland I.J., Accili D. (2014). Integrated control of hepatic lipogenesis versus glucose production requires FoxO transcription factors. Nat. Commun..

[64] El-Brolosy M.A., Stainier D.Y.R. (2017). Genetic compensation: A phenomenon in search of mechanisms. PLoS Genet..

[65] Rouf M.A., Wen L., Mahendra Y., Wang J., Zhang K., Liang S., Wang Y., Li Z., Wang Y., Wang G. (2023). The recent advances and future perspectives of genetic compensation studies in the zebrafish model. Genes Dis..

[66] Shimano H., Shimomura I., Hammer R.E., Herz J., Goldstein J.L., Brown M.S., Horton J.D. (1997). Elevated levels of SREBP-2 and cholesterol synthesis in livers of mice homozygous for a targeted disruption of the SREBP-1 gene. J. Clin. Investig..

[67] Marin-Valencia I., Roe C.R., Pascual J.M. (2010). Pyruvate carboxylase deficiency: mechanisms, mimics and anaplerosis. Mol. Genet. Metab..

[68] Bissonnette B. (2006).

[69] Lacabanne D., Sowton A.P., Jose B., Kunji E.R.S., Tavoulari S. (2025). Current Understanding of Pathogenic Mechanisms and Disease Models of Citrin Deficiency. J. Inherit. Metab. Dis..

[70] Holeček M. (2023). Aspartate-glutamate carrier 2 (citrin): a role in glucose and amino acid metabolism in the liver. BMB Rep..

[71] Fisch S., Gray S., Heymans S., Haldar S.M., Wang B., Pfister O., Cui L., Kumar A., Lin Z., Sen-Banerjee S. (2007). Kruppel-like factor 15 is a regulator of cardiomyocyte hypertrophy. Proc. Natl. Acad. Sci. USA.

[72] Amemiya-Kudo M., Shimano H., Yoshikawa T., Yahagi N., Hasty A.H., Okazaki H., Tamura Y., Shionoiri F., Iizuka Y., Ohashi K. (2000). Promoter Analysis of the Mouse Sterol Regulatory Element-binding Protein-1c Gene. J. Biol. Chem..

[73] Takeuchi Y., Yahagi N., Nakagawa Y., Matsuzaka T., Shimizu R., Sekiya M., Iizuka Y., Ohashi K., Gotoda T., Yamamoto M. (2007). In vivo promoter analysis on refeeding response of hepatic sterol regulatory element-binding protein-1c expression. Biochem. Biophys. Res. Commun..

[74] Yahagi N., Shimano H., Hasty A.H., Matsuzaka T., Ide T., Yoshikawa T., Amemiya-Kudo M., Tomita S., Okazaki H., Tamura Y. (2002). Absence of sterol regulatory element-binding protein-1 (SREBP-1) ameliorates fatty livers but not obesity or insulin resistance in Lepob/Lepob mice. J. Biol. Chem..

[75] Yahagi N., Shimano H., Matsuzaka T., Najima Y., Sekiya M., Nakagawa Y., Ide T., Tomita S., Okazaki H., Tamura Y. (2003). p53 Activation in adipocytes of obese mice. J. Biol. Chem..

[76] Yahagi N., Shimano H., Matsuzaka T., Sekiya M., Najima Y., Okazaki S., Okazaki H., Tamura Y., Iizuka Y., Inoue N. (2004). p53 involvement in the pathogenesis of fatty liver disease. J. Biol. Chem..

