# Supplementary material for: FoxO transcription factors coordinate the urea cycle and gluconeogenesis by controlling Ass1
Source: iScience. 2026 Aug 31;29(9):117399. doi: 10.1016/j.isci.2026.117399 (PMC13562413; doi:10.1016/j.isci.2026.117399)
Supplement: Supplementary file 1 [file mmc1.pdf]

**iScience, Volume *29***

## Supplemental information

# FoxO transcription factors coordinate the urea cycle and gluconeogenesis by controlling *Ass1*

**Samia Karkoutly, Yoshinori Takeuchi, Zahra Mehrazad Saber, Duhan Tao, Tsolmon Mendsaikhan, Rika Saikawa, Yuichi Aita, Yuki Murayama, Akito Shikama, Yukari Masuda, and Naoya Yahagi**

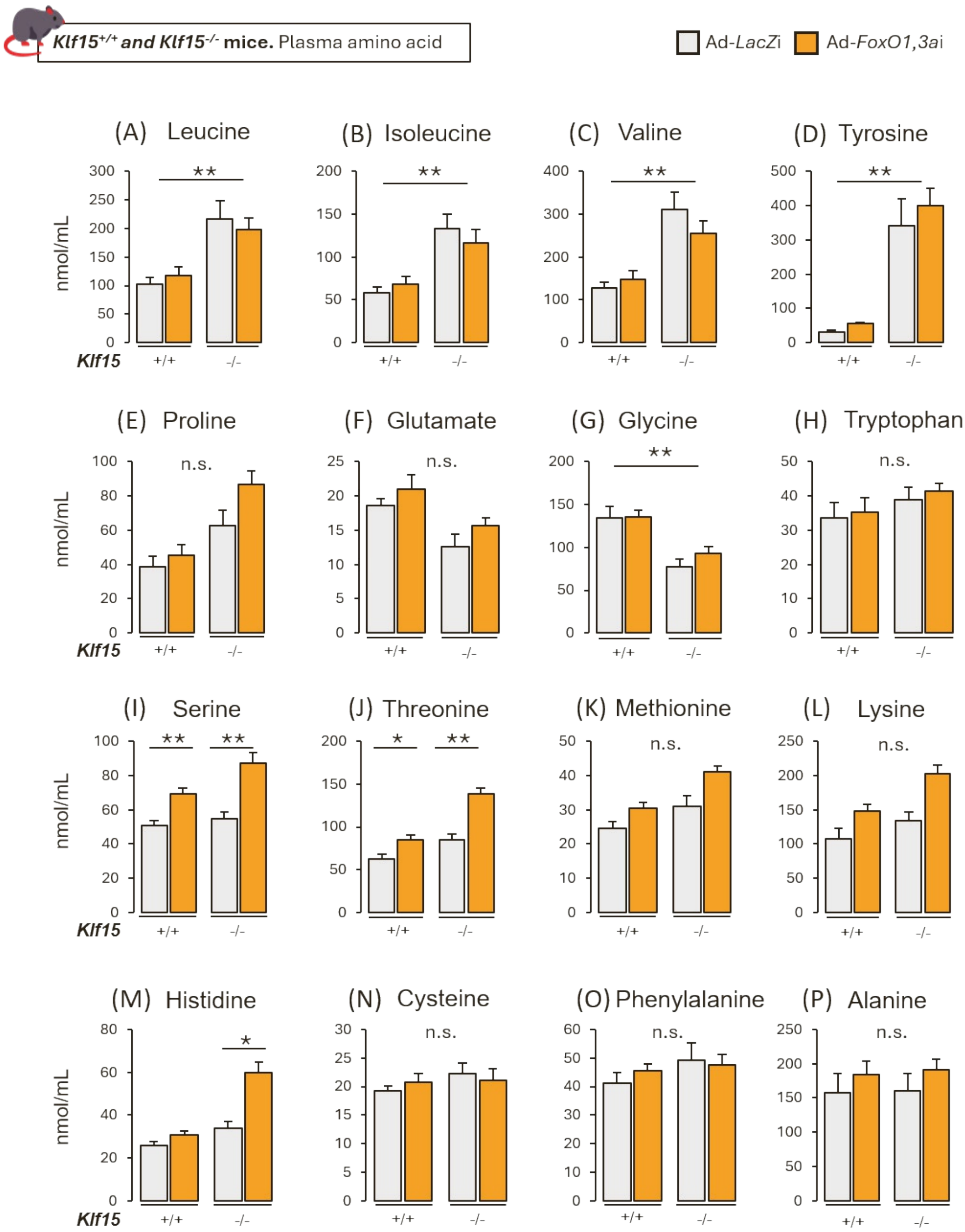


**Figure S1. Plasma amino acids in *Klf15* wild-type and knockout mice. (Related to Figure 1).** Amino acid levels in the plasma of *Klf15* wild-type and knockout C57BL/6J mice with hepatic *FoxO1*/*FoxO3a* knockdown (n = 9-12 per group). *FoxO1* and *FoxO3a* knockdown were performed using adenovirus mediated shRNA (Ad-*FoxO1,3a*i). All groups of mice were sacrificed in the light cycle and blood samples were collected in the fasted state following 24-h fasting. All values are presented as the mean with error bars representing the SEM. For multiple group comparisons, statistical significance was determined by two-way ANOVA followed by Šídák's multiple comparisons test was used. Differences were considered significant if P < 0.05 (*P < 0.05, **P < 0.01).

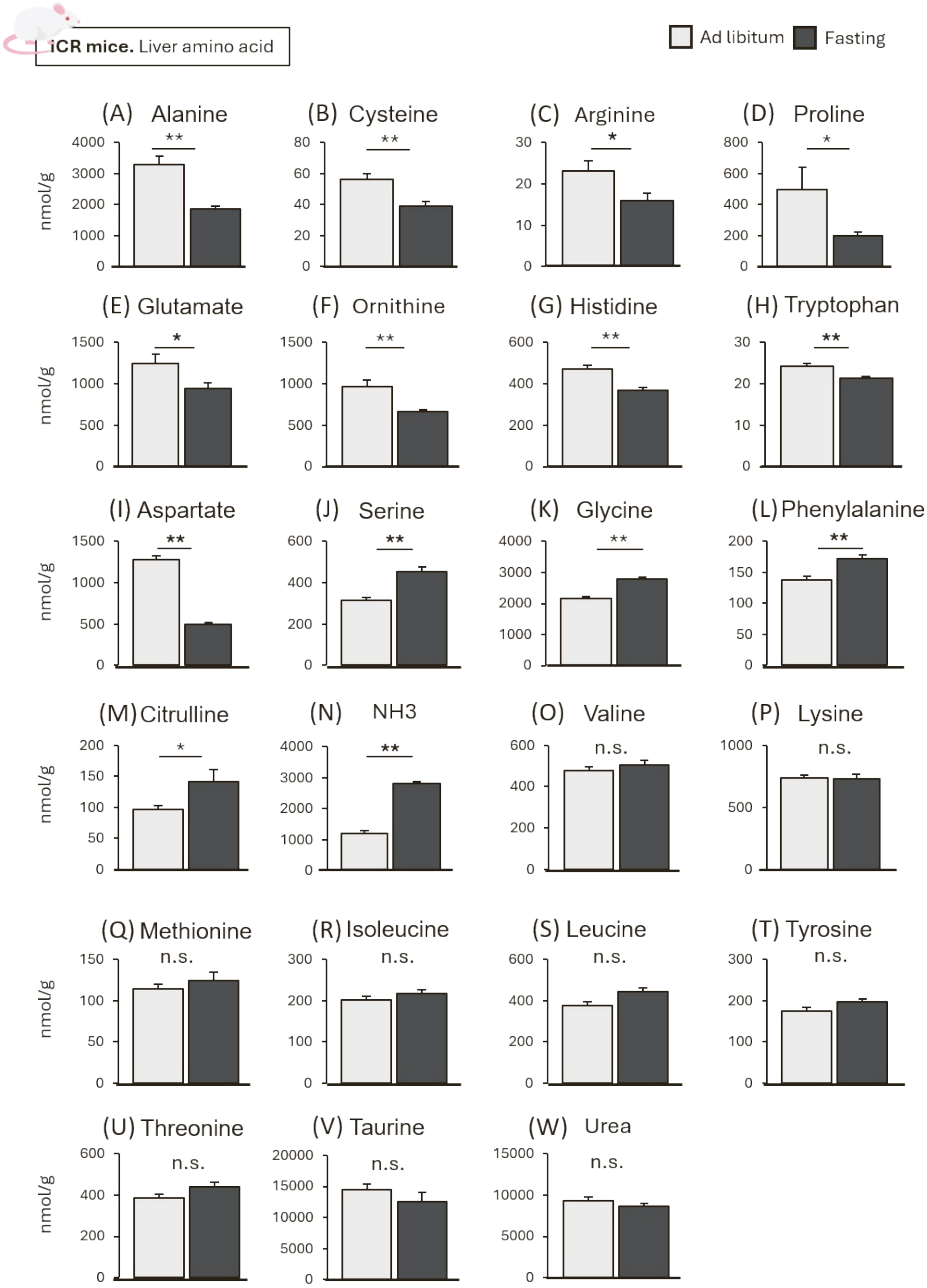


**Figure S2. Hepatic amino acid profile in ICR mice during fasting. (Related to Figure 1).** Amino acid levels in the liver of ICR mice in ad libitum and fasted state (n = 12 per group). All groups of mice were sacrificed in the light cycle and liver samples were collected in the fasted state following 24-h fasting or ad libitum as indicated. All values are presented as the mean with error bars representing the SEM. Datasets were assessed by Student's *t*-test for unpaired samples. The differences were considered to be significant if $P < 0.05$ (*$P < 0.05$ and **$P < 0.01$).

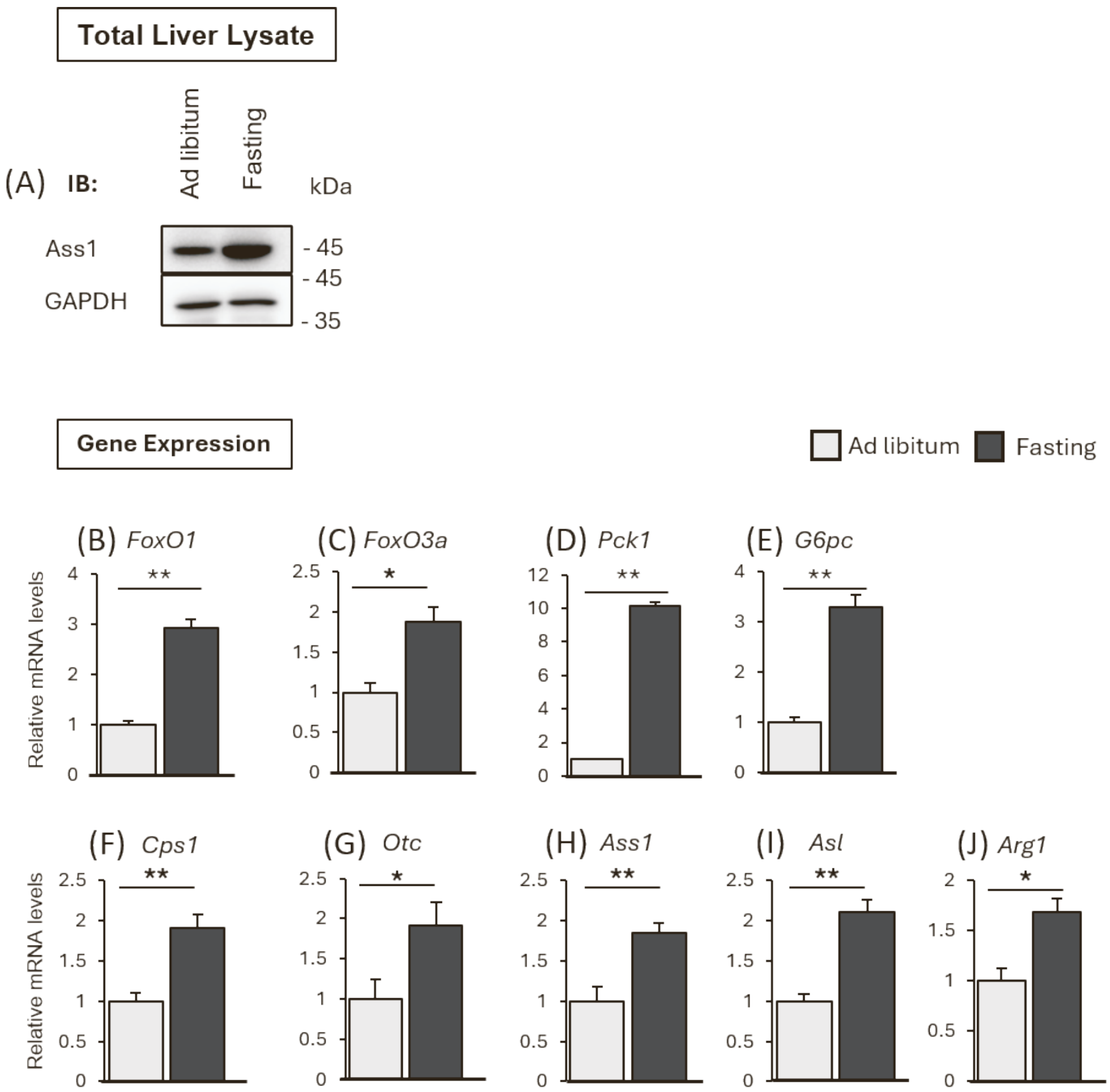


**Figure S3. Effect of fasting on FoxO transcription factors, gluconeogenesis and urea cycle genes. (Related to Figure 2).**

(A) Immunoblot analysis of ASS1 protein using liver total lysate from mice in the fasted and ad libitum state (Samples were pooled from 3 mice). GAPDH was detected as an internal loading control. (B-K) Q-RT PCR analysis of liver RNA samples. Relative gene expression was analyzed in ICR mice in ad libitum and fasted state (n = 9 per group). As the correction of the gene expression level for each sample *Cyclophilin A* was used. All groups of mice were sacrificed in the light cycle and liver samples were collected in and the fasted state following 24-h fasting or ad libitum as indicated. All values are presented as the mean with error bars representing the SEM. Datasets were assessed by Student's *t*-test for unpaired samples. The differences were considered to be significant if $P < 0.05$ (*$P < 0.05$ and **$P < 0.01$).

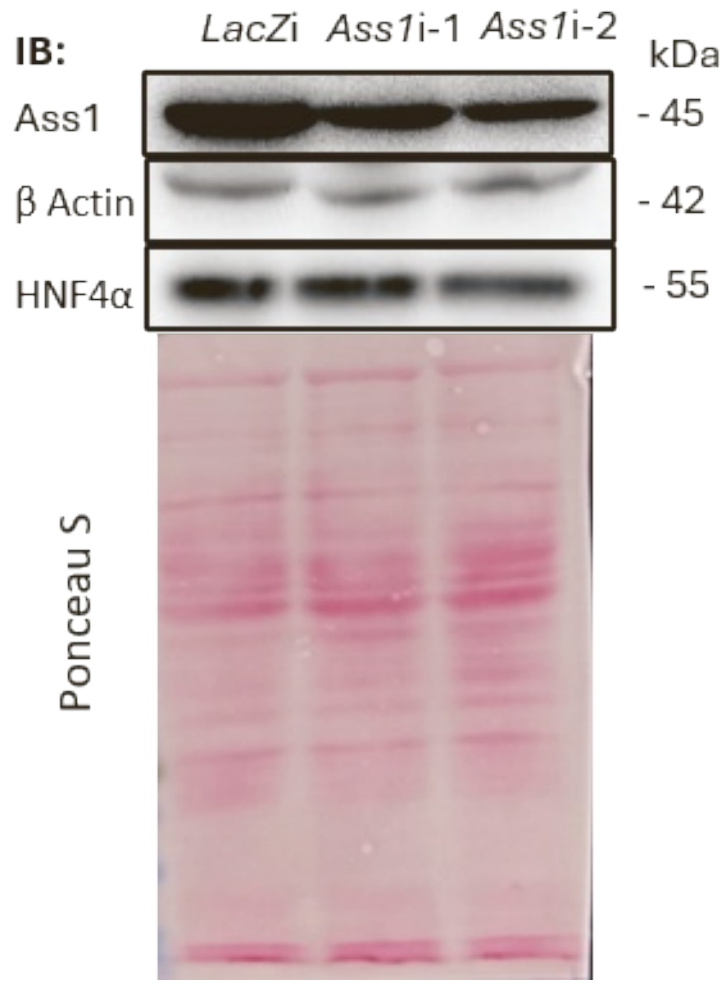


**Figure S4. Alternative internal loading controls. (Related to Figure 3H).**

β-actin (a structural protein unrelated to glucose or nitrogen metabolism) and HNF-4α (a hepatocyte-specific transcription factor confirming cell-type-appropriate protein input) were used as internal loading controls for Figure 3H. Ponceau S staining was additionally performed to provide an antibody-independent assessment of transfer efficiency and loading uniformity across all lanes.

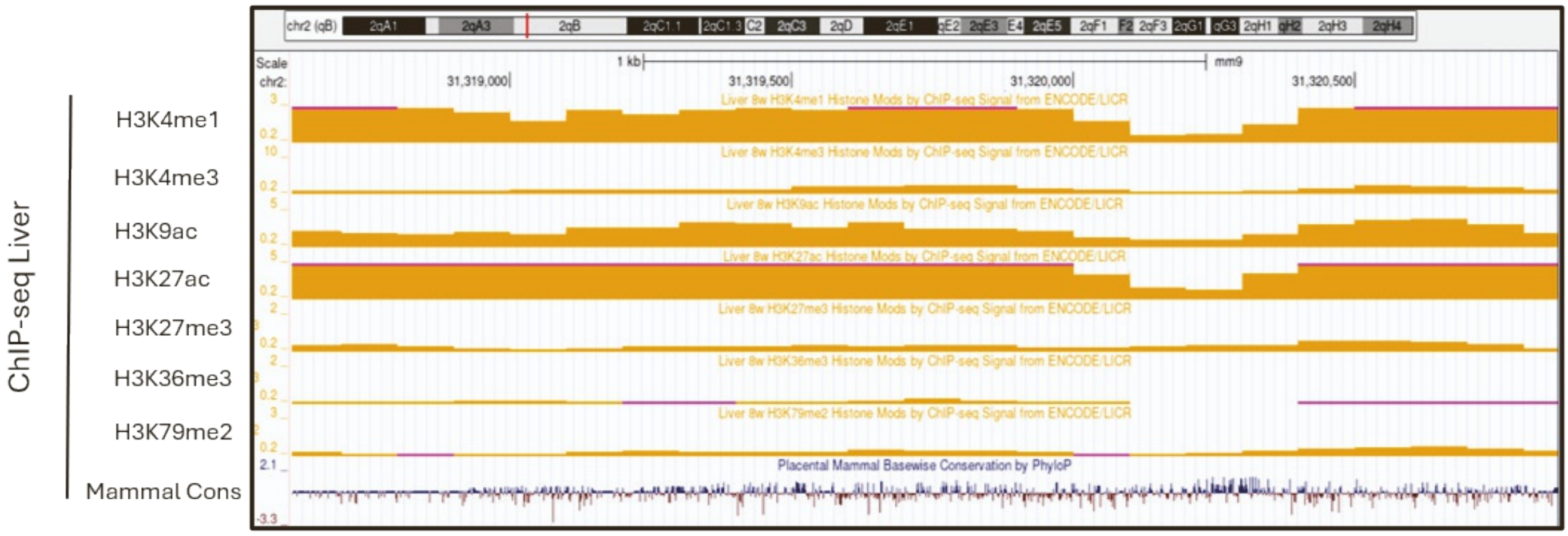


**Figure S5. Genomic information on *Ass1* enhancer. (Related to Figure 5).**
Modified Histones binding regions on *Ass1 enhancer* from ENCODE ChIP-seq database (Liver 8w by ChIP-seq Signal from ENCODE/LICR) on Genome browser. These peaks mean promoter/enhancer regions. Mammalian conservation regions are shown.

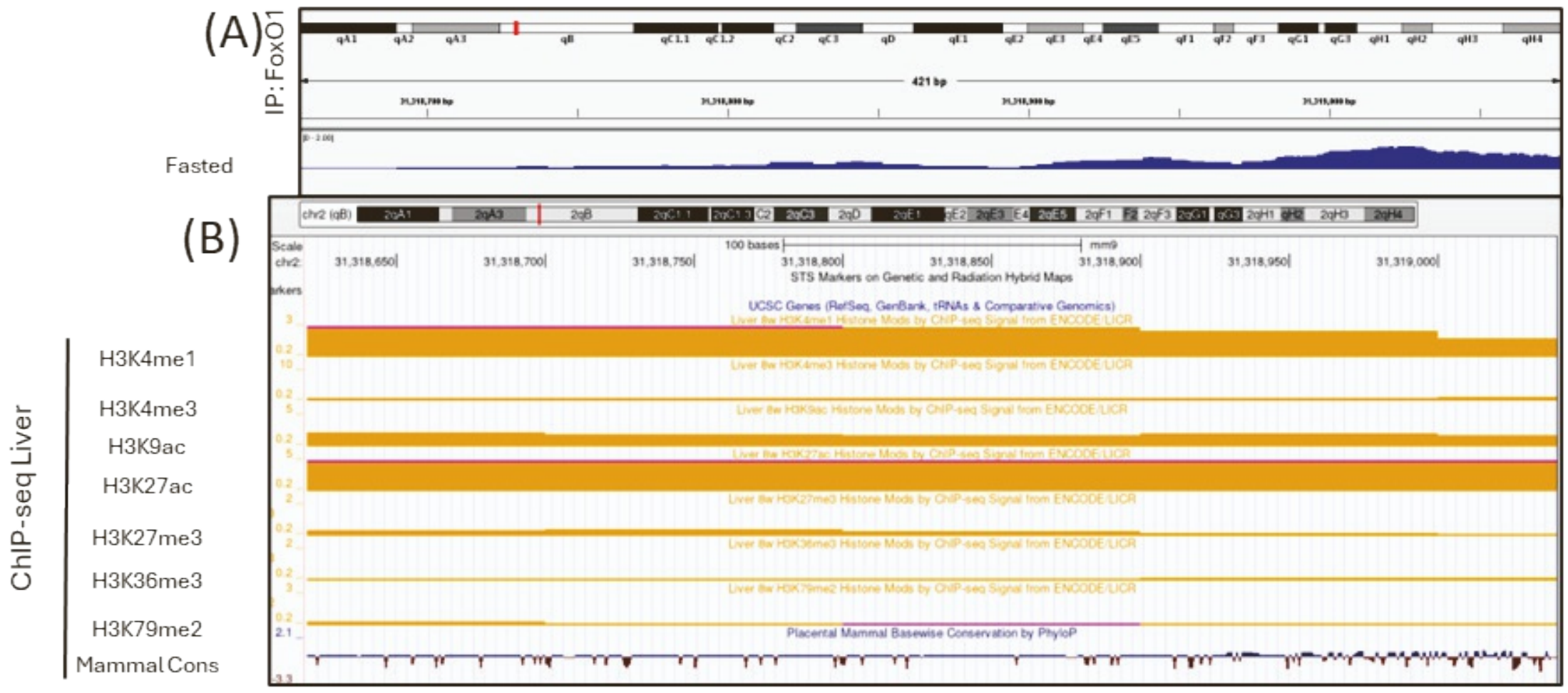


(C) 1A fragment sequence

TCACAATTCTCCCTTGGTCTCCCGAGTGGACCTACAGATGTGCCCCAGCGCTCCTAGA
TATCTAGGCTAGAAGTGGAGTTACAGGTCGTTGTGAAGCACCCGCTGTGTGAATGCTG
GGAATGGAACCCAGGTCCTCTGAAAGTCGCCAGTGCTCTTAACCAGGAGCCGACTCTC
CAACCCTAGATTAGAAGGTACCCATACAGCCAACAGAGTGTACCCAAGGACACATCTA
CTTTCTTTCTGATTTTTGTTTGTTTTGTTGGGTGTTTGTTGAAGTAGGGTCTCACGTA
GCCCAGGATGACCCCACACTCACTGCGTAGCTGAAGATAACCTTGAGTGTTTGCACTC
CAGGCACGTGCCTCCACGTCCCGTCAGCCTTCTACTTTCAAAACAGATTTAAGTGAGT
CCAGGCTTCTG

(D) JASPAR scores for predicated binding sites in 1A fragment

| Matrix ID | Name | Score | Relative score | Strand | Predicted sequence |
|---|---|---|---|---|---|
| MA0480.1 | Foxo1 | 4.4610395 | 0.809847383 | + | TTTTGTTTGTT |
| MA0480.1 | Foxo1 | 4.274477 | 0.807000426 | - | ATCTGTTTTGA |

**Figure S6. Genomic information on FoxO binding site in the *Ass1* enhancer. (Related to Figure 5).**

(A) the results of ChIP-seq with anti-FoxO1 antibody in livers of unfed mice obtained from ChIP-Atlas (ID: GSM3381273) of the smallest fragment 1A. (B) Modified Histones binding regions on *Ass1* enhancer from ENCODE ChIP-seq database (Liver 8w by ChIP-seq Signal from ENCODE/LICR) on Genome browser. These peaks means promoter/enhancer regions. Mammalian conservation regions are shown below ChIP-seq peaks data. (C) Mouse genomic sequence of the smallest fragment 1A. (D) Estimation of FoxOs binding sites on the smallest fragment 1A by JASPAR2020 (http://jaspar.genereg.net/). In red is *Ass1* FoxO BS1, in blue is *Ass1* FoxO BS2 were used as probs for EMSA in Figure 4D and 4E.

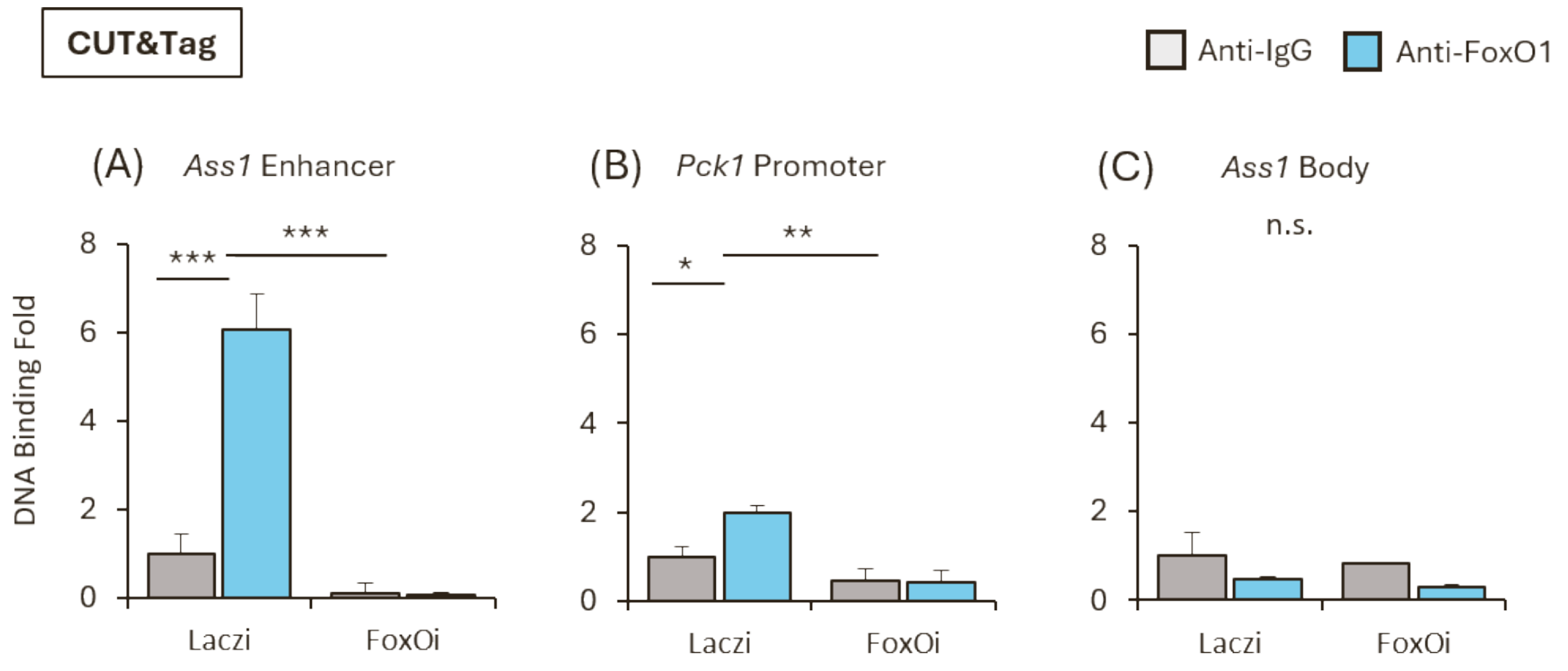


**Figure S7. CUT&Tag assay validates FoxO1 occupancy at the *Ass1* enhancer in mouse liver. (Related to Figure 7).**

CUT&Tag assays were performed on liver samples from ICR mice (6–8 weeks; N = 6 per group) with hepatic *FoxO1/3a* knockdown (Ad-*FoxO1,3a*i) or LacZ knockdown (Ad-*LacZ*i) as control. Mice were fasted for 24 hours before sacrifice. Tagmentation was performed with anti-FoxO1 antibody or normal IgG as a negative control. Locus-specific enrichment was quantified by qPCR using forward primers flanking the validated FoxO-binding site within the *Ass1* enhancer, the *Pck1* promoter FoxO-binding site (positive control locus), and the *Ass1* gene body (within-locus negative control), all paired with a common i7 reverse primer to ensure selective detection of tagmented, antibody-captured fragments. Data are expressed as fold enrichment over IgG. Anti-FoxO1 enrichment at the *Ass1* enhancer and *Pck1* promoter was significantly higher in Ad-*LacZ*i controls compared to Ad-*FoxO1,3a*i mice, and was markedly abolished upon *FoxO1/3a* depletion. No significant enrichment was detected at the *Ass1* gene body in either group. Data represent mean ± SEM. For multiple group comparisons, statistical significance was determined by two-way ANOVA followed by Šídák's multiple comparisons test. Differences were considered significant if $P < 0.05$ (*$P < 0.05$, **$P < 0.01$, *** $P < 0.001$).

**Table S1. List of primer sets used for Q-RT PCR.**

| Gene (mouse) | Sequences for Q-RT PCR primers |
|---|---|
| ***Ass1*** | 5′-TGGCCAGAAGGAAGACTTTG-3′<br>5′-GACAGCAGGCCAGATGAA-3′ |
| ***FoxO1*** | 5′-TTCAATTCGCCACAATCTGTCC-3′<br>5′-GGGTGATTTTCCGCTCTTGC-3′ |
| ***FoxO3a*** | 5′-TGTGCCCTACTTCAAGGATAAG-3′<br>5′-TTCATTCTGAACGCGCAT-3′ |
| ***Pck1*** | 5′-TGTCATCCGCAAGCTGAAGA-3′<br>5′-TTCGATCCTGGCCACATCTC-3′ |
| ***G6pc*** | 5′-GGGCGCAGCAGGTGTATACTAT-3′<br>5′-CAGAATCCCAACCACAAGATGA-3′ |
| ***Arg1*** | 5′-GGAGGCCTATCTTACAGAGAAGGTC-3′<br>5′-CGAAGCAAGCCAAGGTTAAAGCCAC-3′ |
| ***Cps1*** | 5′-GACTTCTCCAGACCTGGCAT-3′<br>5′-TCCTCTGTTGCTGGTGAAGTG-3′ |
| ***Otc*** | 5′-TACATGCTATGGCTCTCTGCA-3′<br>5′-TCTGTGGACAGTCTTGTTCGA-3′ |
| ***Asl*** | 5′-CCCAAGGCACCTTCAAAT-3′<br>5′-GCGACTTCGTCCTGTGTGTA-3′ |
| ***Cyclophilin B*** | 5′-TGGCTCACAGTTCTTCATAACCA-3′<br>5′-ATGACATCCTTCAGTGGCTTGTC-3′ |
| ***Gapdh*** | 5′-TGTGTCCGTCGTGGATCTGA-3′<br>5′-CACCACCTTCTTGATGTCATCATAC-3′ |

**Table S2. List of primer sets used for PCR.**

| Ass1-enhancer-Luc | Sequence for PCR |
|---|---|
| **Full** | 5′-TACGCGTGCTAGCCCCGAGTGGACCTACAGATGTGC-3′<br>5′-GCAGATCTCGAGCCCGCCAGGTGTCTCTTGCATGTAG-3' |
| **Fr1** | 5′-TACGCGTGCTAGCCCCGAGTGGACCTACAGATGTGC-3′<br>5′-GCAGATCTCGAGCCCGACTTAAAGCAGAGGTG-3′ |
| **Fr2** | 5′-TACGCGTGCTAGCCCACCTCTGCTTTAAGTC-3′<br>5′-GCAGATCTCGAGCCCACATGCTTACCTCTTAGC-3′ |
| **F3** | 5′-TACGCGTGCTAGCCCGCTAAGAGGTAAGCATGT-3′<br>5′-GCAGATCTCGAGCCCGCCAGGTGTCTCTTGCATGTAG-3' |
| **Fr1A** | 5′-TACGCGTGCTAGCCCCGAGTGGACCTACAGATGTGC-3′<br>5′-GCAGATCTCGAGCCCCCTTTACGAAGAAGTGTGC-3′ |
| **Fr1B** | 5′-TACGCGTGCTAGCCCGCACACTTCTTCGTAAAGG-3′<br>5′-GCAGATCTCGAGCCCGACTTAAAGCAGAGGTG-3′ |
| **Mutated** | 5′-GATTTTGGATCCTTTGTTGGGTGTTTGTTGA-3′<br>5′-CAACAAAGGATCCAAAATCAGAAAGAAAGTA-3′ |

**Table S3. List of EMSA probes.**

| Probe | Sequence |
|---|---|
| ***Pck1*** | 5′-AGCTGTGGTGTTTTGACAAC-3′<br>5′-GCTGGTTGTCAAAACACCA-3′ |
| **Binding Site1** | 5′-GTGTACCCAAGGACACATCTACTTTCTTTCTGATTTTTGTTTGTTTT-3′<br>5′-CACCCAACAAAACAAACAAAAAT-3′ |
| **Binding Site2** | 5′-GTCCCGTCAGCCTTCTACTTTCAAAACAGATTTA-3′<br>5′-GGACTCACTTAAATCTGTTTTGAAA-3′ |
| **Binding Site1 mut** | 5′-GTGTACCCAAGGACACATCTACTTTCTTTCTGATTTTGGATCCTTT-3′<br>5′-CACCCAACAAAGGATCCAAAAT-3′ |

**Table S4. List of primer sets used for ChIP assay.**

| Gene (mouse) | Sequences for ChIP Q-PCR primers |
|---|---|
| *Ass1* Enhancer | 5′-CAGGAGCCGACTCTCCAACC-3′<br>5′-CAGCTACGCAGTGAGTGTGG-3′ |
| *Ass1* Body | 5′-TTGCAGGCCGCAGACACCA-3′<br>5′-GGATCTTGGCTGGCCTGGA-3′ |
| *Arg1* Promoter | 5′-GCAATGGGCATTCTGCACCC-3′<br>5′-CTTGGTGTTACCGGCTGGC-3′ |
| *Pck1* Promoter | 5′-CCCAGCTAACTCAGCAAGGTAC-3′<br>5′-GATTGACTAAACTTTGACCCTGC-3′ |
| *G6pc* Promoter | 5′-GCCTCTAGCACTCAAGCAGTG-3′<br>5′-TGTGCCTTGCCCCTGTTTTATATG-3′ |

**Table S5. List of primer sets used for CUT&Tag assay.**

| Gene (mouse) | Sequences for CUT&Tag Q-PCR primers |
|---|---|
| *Ass1* Enhancer | forward: 5′-CCCATACAGCCAACAGAGTG-3′ |
| *Ass1* Body | forward: 5′-TTCTCATGACCTTTGGCCGT-3′ |
| *Pck1* Promoter | forward: 5′-TTGCAGGCCGCAGACACCA-3′ |
